# Effects of DL-Methionine or Methionine Hydroxy Analogue-Free Acid Supplementation on Growth Performance and Carcass Yield of Broilers Fed Reduced Energy Diets with Guanidinoacetic Acid Supplementation

**DOI:** 10.3390/ani16121811

**Published:** 2026-06-11

**Authors:** Patrícia Tomazini Medeiros, Andreas Lemme, Victor Naranjo, Mariane Possamai, Rafael Rancatti, Alex Maiorka

**Affiliations:** 1 Veterinary Sciences, Federal University of Paraná, Curitiba 80035-050, PR, Brazil; amaiorka@ufpr.br; 2 Evonik Operations GmbH, 63457 Hanau, Germany; andreas.lemme@evonik.com; 3 Evonik Guatemala, Guatemala City 01010, Guatemala; victor.naranjo@evonik.com; 4 Vibra Foods, Montenegro 92521-000, RS, Brazil; mariane.della@vibra.com.br; 5 Vibra Foods, Pato Branco 85503-378, PR, Brazil; rafael.rancatti@vibra.com.br

**Keywords:** broilers, DL-methionine, methionine hydroxy analogue, guanidinoacetic acid, dietary energy, growth performance, feed conversion, carcass yield

## Abstract

Guanidinoacetic acid (GAA) and methionine are two biologically active compounds related to cell energy metabolism. Both are metabolically linked, as the methyl group needed to transform GAA into creatine derives from S-adenonsylmethionine out of the homocysteine cycle. A broiler feeding study was conducted to evaluate potential interactions between 600 g/t supplemental GAA and feed additives with methionine activity. The experimental design comprised two parts. Part one consisted of three dietary treatments in which DL-methionine (DL-Met) was added to meet sulfur amino acid recommendations (1), DL-Met was replaced with liquid methionine hydroxy analogue-free acid (MHA-FA) assuming an 80% methionine activity in the product (2), and DL-Met was added at 65% of the MHA-FA dose used in Treatment 2 (on a product weight basis). Treatments 1, 2, and 3 reflect the range of commercial practice with assuming bioefficacies of either 80% (Trt 2 vs. 1) or 65% (Trt 3 vs. 2) for MHA-FA relative to DL-Met. Part two followed a 2 × 4 factorial design. Either DL-Met or MHA-FA (assuming 80% methionine activity) was added to diets with gradually reduced apparent metabolizable energy (AME; standard, −25, −50, −75 kcal/kg AME). For MHA-FA, a bioefficacy of 80% relative to DL-Met was applied in order to evaluate interactions with GAA efficiency assuming inadequate dietary Met+Cys supply with MHA-FA. AME-reduced diets were supplemented with 600 g/t GAA to evaluate its energy-sparing potential. A total of 2784 male Cobb 500 broilers were equally assigned to 116 floor pens, resulting in 13 replicates (one treatment with 12) per treatment. A five-phase feeding program was introduced from 1 to 40 days of age. Body weights (BW) and feed intake (FI) were recorded in weekly intervals allowing for calculating weight gain (BWG) and feed conversion ratio (FCR). At termination of the experiment, three birds per pen were obtained for carcass analysis. The results revealed that an overestimation of the bioefficacy of MHA-FA relative to DL-Met significantly compromised broiler growth (*p* < 0.05), while applying a bioefficacy of 65% for MHA-FA relative to DL-Met65 resulted in similar performance. However, on day 40, BW of broilers fed DL-Met65 were higher than with MHA-FA (*p* < 0.05). It is concluded that an overestimation of the bioefficacy of MHA-FA relative to DL-Met bears the risk of compromised broiler performance while a bioefficacy of 65% allows at least similar, potentially even better, performance and economic advantages. There were basically no interactions between MHA-FA or DL-Met and graded ME levels with concomitant GAA supplementation regarding overall performance of broilers. This means that final BW of DL-Met-fed broilers were higher than those of MHA-FA broilers independent of dietary ME (*p* < 0.05). In early feeding phases, interactions indicated that broiler performance (BW day 7, 21, *p* < 0.05) diminished with dietary energy reduction with DL-Met supplementation while this was not the case with MHA-FA-fed broilers. However, with standard AME, DL-Met outperformed MHA-FA (*p* < 0.05). Basically, GAA supplementation compensated AME reduction up to 50 kcal/kg as there were no differences in BW, BWG, FCR, FI, or breast meat yield. Additionally, −75 kcal/kg AME was compensated by GAA only up to a broiler age of 21 days.

## Simple Summary

An experiment was conducted to investigate the effects of guanidinoacetic acid (GAA) added to experimental diets, which were gradually reduced in metabolizable energy (AME) content, and the use of either DL-methionine (DL-Met) or the liquid methionine hydroxy analogue-free acid (MHA-FA) as methionine. The latter thus were potential methyl-group donators for metabolic transformation of GAA into creatine. The results indicated that an overestimation of the bioefficacy of MHA-FA relative to DL-Met compromises broiler performance while applying a bioefficacy of 65% for MHA-FA relative to DL-Met allows for similar performance but economic advantages. Basically, an overestimation of the relative bioefficacy of MHA-FA did not interact with the efficiency of GAA supplemented to energy-reduced diets, although in earlier feeding phases, DL-Met-fed broilers were more affected by AME reduction than MHA-FA-fed broilers. However, the responses of the broilers suggest that a reduction in AME up to 75 kcal/kg can be compensated by 600 g/t GAA without affecting broiler performance up to 21 days of age, while in older birds, the AME-sparing potential of GAA was 50 kcal/kg.

## 1. Introduction

Poultry production holds a central role in the global supply of high-quality protein, driven by the continuous growth in worldwide demand for chicken meat. Projections indicate that the global population will surpass 9.7 billion by 2050, reinforcing the need for sustainable and efficient production systems [1]. In this context, broiler chickens stand out due to their high growth rate and excellent feed conversion efficiency, requiring increasingly precise nutritional strategies to optimize performance and health [2,3].

With respect to broiler feed production, dietary energy has been reported to represent 75% of the total cost of feed [4]. Therefore, both the scientific community and the commercial world investigated many strategies to optimize dietary energy utilization and diet prices. Strategies include optimized use of oil, carbohydrate, and protein sources, but also feed additives such as enzymes or concepts for dietary energy assessment [4]. Researchers, thus, also compared the effectiveness of various feed additives including NSP-degrading enzymes, emulsifiers, or guanidinoacetic acid (GAA) showing that a supplementation of 600 g/t could compensate for reductions in dietary AME by 50 up to 200 kcal/kg [5,6]. Accordingly, supplementation of 600 g/t GAA to diets reduced by 50 kcal AME/kg have shown significant reductions in feed cost per gain by 11% [5] and improvements in profitability of broiler production by 6% [7], demonstrating the economic relevance.

Guanidinoacetic acid has gained increasing attention due to its physiological role as the direct precursor for creatine formation, an essential molecule for muscle energy metabolism [8,9]. While GAA is completely digestible and, thus, fully available for metabolic purposes [8,10], it also increases muscle creatine as well as phosphorylated creatine content [9,11,12,13,14]. Phosphocreatine accumulation enhances the cellular energy status and exerts an arginine-sparing effect, allowing this amino acid to be directed towards other metabolic and immunological functions [9,15,16]. Recent studies have shown that dietary supplementation with GAA can improve growth performance, carcass yield, antioxidant capacity, and intestinal integrity in broilers, particularly under challenging conditions such as low-energy diets or heat stress [9,17]. One side effect of an improved cell energy metabolism is a beneficial effect of GAA supplementation on reducing meat myopathies [18,19].

Although robust evidence supports the benefits of GAA, variability regarding productive and physiological responses among studies still exists—likely influenced by factors such as dosage, bird age, dietary energy level, or environmental conditions [9,12,20,21]. Another factor that may interact with GAA is the choice of the feed additive with methionine activity, since the conversion of GAA into creatine in the liver requires methylation via S-adenosyl-methionine, indicating that dietary methionine is a key nutritional factor influencing GAA efficiency [22].

Numerous studies have reported that the bioefficacy of liquid hydroxy analogue of methionine free acid (MHA-FA; 88% purity) is approximately 65% relative to that of DL-methionine (DL-Met; 99% purity) on a weight basis [23,24]. Other studies suggest values closer to 80% [25,26]. Both estimates are applied in commercial practice. However, the application of an inappropriate bioefficacy for MHA-FA can lead to compromised growth of broilers, increased feed conversion ratio, and impaired economic outcomes [23,24]. Therefore, in this study, both the strategies assuming 80% relative bioefficacy and assuming 65% relative bioefficacy of MHA-FA were considered. Moreover, inadequate dietary supply with dietary Met+Cys as a result of an overestimation of the relative bioefficacy of MHA-FA might interact with creatine metabolism specifically with the methylation of GAA [22]. The methylation of GAA to form creatine is catalyzed by the N-guanidinoacetate methyltransferase and takes place in the liver [9,17,22]. It has been estimated that creatine synthesis accounts for at least 70% of the total utilization of methyl groups [22]. Wyss and Kaddurah-Daouk [22] speculate that the provision of methyl groups should not become a limiting factor for creatine formation under normal nutritional conditions, but they also see a limitation potential in the case of impaired S-adenosyl-L-methionine synthesis. Klünemann et al. [27] reported that genes related to sulphur amino acid metabolism and cysteine formation from homocysteine in the liver were strongly upregulated in the case of dietary Met+Cys deficiency compared to adequate supply. It is concluded that in the case of marginal dietary methionine supply or a methionine deficiency, the remethylation of homocysteine to methionine can be limited. In a high methionine supply status, homocysteine will enter the transsulfuration pathway to a larger extent while at deficiency, the remethylation pathway to form methionine is preferred [28]. Accordingly, when halving the dietary methionine supply in human or rat nutrition, the number of remethylation cycles doubled to about 3–4 cycles [28]. These findings also imply that the number of remethylation cycles is not unlimited and thus is dependent on dietary methionine supply. While the addition of two equimolar levels (0.03%, 0.09%) of either DL-Met or MHA-FA to broiler diets reduced the remethylation of homocysteine to the same magnitude, body weight gain was significantly increased [29]. However, at 0.03% supplementation, body weight gain was significantly lower with MHA-FA compared to DL-Met, suggesting a limited dietary Met+Cys supply with MHA-FA. On the other hand, in a methionine-deficient diet, 600 g/t GAA supplementation failed to improve the feed conversion ratio of broilers in contrast to both equimolar creatine monohydrate supplementation to the methionine-deficient diet and GAA and creatine monohydrate supplementation to diets adequate in methionine [30]. It is suggested that this was because of a limitation of homocysteine remethylation capacity.

Therefore, the current study comprised two parts. One part aimed to investigate the impact of two assumed bioefficacies for MHA-FA of either 80% or 65% relative to DL-Met on broiler performance as well as to test whether the concept of 65% bioefficacy for MHA-FA relative to DL-Met allows for the same broiler performance. The objective of the second part of the study was to evaluate interactions between the efficiency of supplemental GAA as an energy-sparing feed additive and either DL-Met or MHA-FA. While the source of methionine products may impact the methyl group donation capacity for creatine formation, graded reductions in dietary AME along with GAA supplementation were introduced to evaluate the energy-sparing potential of GAA.

## 2. Materials and Methods

### 2.1. Ethical Approval and Animal Care

All experimental procedures involving animals were approved by the Ethics Committee on the Use of Animals of the Campus of Agricultural Sciences, Federal University of Paraná (approval date: 28 March 2025). The study was conducted in accordance with the ethical guidelines for the use of live poultry in research established by the Brazilian College of Federal University of Parana State.

### 2.2. Animals and Housing

The study was conducted at a Poultry Research Center located in Pato Branco, PR, Brazil. A total of 2784 day-old male Cobb 500™ chicks, originating from 40-week-old breeders and with an initial body weight of 45.04 g (±0.37), were obtained from the company’s hatchery located in Coronel Vivida, PR, and used in this study.

The birds were housed in floor pens, 116 boxes ([1.85 m^2^], with 13 birds/m^2^ equipped with tubular feeders and nipple drinkers (three nipples per pen) and bedded with wood shavings (litter reused for the fifth time). Feed and water were provided *ad libitum* during the study period. Lighting was maintained according to the company’s Program Light Guidance using high light intensity (20–25 lux) during the first week and gradually reducing it to about 5 lux, following Cobb’s recommendation [31]. At the hatchery, all birds received the following vaccinations according to commercial practices: Marek’s disease, Gumboro disease, Fowlpox, and Infectious Bronchitis.

### 2.3. Experiment Design

The feeding trial included 9 treatments listed in Table 1:

The study design consisted of two parts. Part A included three dietary treatments differing only in dose and choice of either DL-Met or the methionine precursor MHA-FA. It was hypothesized that Treatment 2 would result in a lower broiler performance than Treatment 1, whereas broiler performance of Treatment 3 would not differ from that of Treatment 2. This hypothesis is based on two consecutive meta-analyses and literature reviews on the differentiation of DL-Met and MHA-FA as feed supplements [23,24]. On the one hand, simultaneous dose–response trials revealed an average bioefficacy of 63% for MHA-FA relative to DL-Met, which, per definition, implies that applying a bioefficacy of 80% for MHA-FA would result in lower performance compared to DL-Met-fed broilers particularly with marginal dietary Met+Cys levels [23]. On the other hand, numerous broiler feeding trials conducted under various nutritional and management conditions tested the recommended relative bioefficacy concept of 65% relative bioefficacy for MHA-FA by replacing supplemental MHA-FA with 65% as much as DL-Met. The meta-analysis revealed that the body weight gain and feed conversion ratio of broilers did not differ between these two dietary treatments in any case [24].

Part two was designed as a 4 × 2 factorial arrangement, comprising four dietary metabolizable energy (AME) levels (standard or reduced by 25, 50, or 75 kcal/kg) and two feed additives to balance dietary Met+Cys (DL-Met or MHA-FA). In addition, diets with reduced AME were supplemented with 600 g/t GAA. The rationale behind the decision was that GAA has been reported to have a AME-sparing effect in broiler diets [9]. Accordingly, performance of broilers fed energy-reduced diets supplemented with GAA would not differ from performance achieved with standard feeds. A dose–response approach with graded dietary AME reductions would allow for assessing up to which dietary AME reduction GAA can maintain broiler performance. It should be noted that standard diets without GAA serve as control because energy sparing is not applicable. As GAA needs methyl groups deriving from dietary methionine via the homocysteine cycle, a possible interaction with DL-Met or MHA-FA was assumed.

Birds were distributed in a completely randomized design with 13 replicate pens per treatment and 24 birds per replicate. Treatment 1 (control, Table 1) included 12 replicates only because of the limitation of the overall capacity of 116 available pens. Birds were fed in 5 feeding phases, pre-starter (day 1–7), starter (day 8–14), grower (day 15–24), finisher I (day 25–35), and finisher II (day 36–40). All diets were pelleted, with the pre-starter and starter diets provided in a crumbled form. For diets with reduced AME levels, GAA was supplemented at 600 g/t of feed in each phase, reflecting common commercial practice when feeding energy-reduced diets with GAA. Standard diets were not supplemented with GAA. The experimental diets were produced in the company’s feed mill located in Itapejara D ’Oeste, PR, Brazil.

Within each dietary AME level, either DL-Met or MHA-FA was included in the diets to meet the Met+Cys requirements for broilers according to the company’s nutritional levels. MHA-FA was included based on 80% of the bioefficacy of DL-Met. Therefore, dietary DL-Met additions were 80% as high as MHA-FA supplementations in corresponding treatments on a product weight basis. Apart from the AME levels, all diets were formulated to meet the Cobb Vantress nutrition recommendations [31]. The feed formulations and the analyzed nutrients are presented in Supplementary Materials, Tables S1–S5 and Tables S6–S10, respectively.

### 2.4. Growth and Slaughter Performance

Body weights (BW) of birds were measured at 1, 7, 14, 21, 28, 35, and 40 days of age to determine body weight gain (BWG). Feed intakes (FIs) were recorded per pen on days 7, 14, 21, 28, 35, and 40. The feed conversion ratio (FCR) was calculated accordingly using FI and BWG and was corrected with mortality. For FCR correction, FI was related to final broiler BW plus BW of dead broilers. On day 40, three birds per pen were selected and slaughtered at the company’s processing plant to measure carcass weight, breast meat weight, and thigh weight. Weights of cuts were related to liveweights of selected birds to calculated yields. All mortalities and their causes were recorded immediately upon observation. A necropsy was performed when the cause of death was not clear. Birds showing compromised health—such as lameness, clinical signs of disease, or severe injury—were humanely culled and recorded.

### 2.5. Statistical Analysis

Statistical analyses were performed with R software (version 4.3.3).

Data of Part A including three dietary treatments were analyzed by one-way analysis of variance (ANOVA) according to the following model:

Y_ij_ = µ + α_i_ + ε_ij_

in which Y_ij_ is the dependent variable, µ is the overall mean, α_i_ is the effect of treatment (i = 1, 2, 3), and ε_ij_ is the residual error. Comparison of means was performed according to Tukey and considering *p* < 0.05 as significant.

Results of Part B of the experiment were analyzed by two-way ANOVA according to the following model:

Y_ijk_ = µ + α_i_ + β_j_ + (αβ)_ij_ + ε_ijk_

in which Y_ijk_ is the dependent variable, µ is the overall mean, α_i_ is the effect of methionine source (j = 1, 2), β_j_ is the effect of dietary energy level (j = 1, 2, 3, 4), (αβ)_ij_ is the interaction between methionine source and dietary energy level, and ε_ijk_ is the residual error. Comparison of means was performed according to Tukey and considering *p* < 0.05 as significant.

Before statistical analysis, data were checked for outliers. Outliers were defined as observations with a z-score larger than ±2, following the empirical rule that approximately 95% of normally distributed data lie within two standard deviations of the mean (NIST; empirical rule [32]). Therefore, data within treatment per performance parameter was removed from statistical analysis when deviating ± two standard deviations of treatment means. Accordingly, one replicate of Treatment 1 (BW day 21, FCR 1–7 days, 2 birds carcass analysis), Treatment 2 (BWG from 15 to 21 days, feed consumption 1–7 days, FCR 8–14 days, FCR 15–21 days, FCR 22–28 days, FCR 1–40 days), Treatment 3 (FCR 1–40 days, 1 bird carcass analysis), Treatment 4 (BWG from 22 to 28 days, feed consumption 15–21 days, FCR 15–21 days, 1 bird carcass analysis), Treatment 5 (1 bird carcass analysis), Treatment 6 (FCR 1–7 days, FCR 1–21 days, 1 bird carcass analysis), Treatment 7 (1 bird carcass analysis), Treatment 8 (FCR 1–7 days), and Treatment 9 (BWG from 22 to 28 days, FCR 22–28 days, FCR 1–28 days, 1 bird carcass analysis) were removed.

In the result tables, data are expressed as means ± standard error of means (SEM).

## 3. Results

### 3.1. Feed Analysis

Feeds were analyzed for nutrients including total amino acids and free methionine, basically representing supplemented DL-Met and MHA-FA (Supplementary Materials, Tables S6–S10). Compared to the expected concentrations, the recovery of GAA in the experimental feeds was consistent across treatments and phases and averaged 97%. The supplementation of MHA-FA was similar between treatments, and the recovery was on average 85%. This means that about 0.05% lower than anticipated MHA-FA levels were analyzed in the samples. Similarly, DL-Met recovery was also on average 84%, equaling about 0.04% lower than planned supplementation levels. Consequently, the resulting ratios between MHA-FA (product) and DL-Met was on average 80% in respective pairs of treatment, which exactly meets the expectations. The ratio DL-Met65 to MHA-FA was 62% on average and confirms, thus, the targeted ratio of 65%. There was one exception: the recovery of DL-Met in all respective samples of finisher I diets was only 55%, which means that it was 0.10% lower than expected. Therefore, the product ratio of DL-Met to MHA-FA was 53% on average and, thus, 27–points lower than anticipated. At the same time, as much as 135% of expected free lysine was found on average in the DL-Met treatments, whereas for the MHA-FA treatments, an average of 87% was reported for this phase.

### 3.2. Methionine Product Comparison

#### 3.2.1. Growth Performance and Feed Conversion Ratio

Results on body weights are shown in Table 2. Body weights differed significantly between treatments at days 7, 14, and 21 (*p* < 0.05). Accordingly, broilers of treatment DL-Met were heavier than broilers of treatment MHA-FA (*p* < 0.05), whereas BW of birds fed DL-Met65 did not differ from the other treatments. On day 40, broilers of both DL-Met and DL-Met65 treatments were heavier than MHA-FA-fed broilers (*p* < 0.05). Weight gains of DL-Met-fed broilers were higher than MHA-FA-fed broilers in the first and the second experimental weeks (*p* < 0.05), whilst BWG of DL-Met65-fed birds were not different compared to the other treatments.

There were no significant treatment effects on FI nor on FCR except for non-corrected FCR over the entire experimental period (Table 2 and Table 3). Accordingly, the FCR of both DL-Met treatments was 3 points lower compared to the MHA-FA treatment (*p* < 0.05). Mortality ranged between 3.7 and 6.5%.

#### 3.2.2. Carcass Evaluation

The carcass evaluation revealed treatment differences in liveweights of selected birds as well as in carcass and breast meat weights (*p* < 0.05). Liveweights of broilers of the DL-Met treatment was significantly higher than that of the other treatments (*p* < 0.05), which did not differ among each other. Carcass and breast meat weights of broilers from the DL-Met65 treatment were significantly lower than weights of broilers of the DL-Met treatment (*p* < 0.05); however, the weights reported for the MHA-FA treatment were in between and did not differ from the DL-Met treatments (Table 3).

### 3.3. Dietary Energy Reduction and Supplementation with 600 g/t Guanidinoacetic Acid Versus Methionine Products (Part B)

#### 3.3.1. Growth Performance and Feed Conversion Ratio

As shown in Table 4, significant effects and interactions between energy reduction and methionine supplements were detected. From day 7 until day 40, BW of DL-Met-fed broilers were significantly higher than BW of MHA-FA-fed broilers (*p* < 0.05). The gradual reduction in dietary AME along with supplementation of 600 g GAA/t in AME-reduced diets affected BW particularly at days 35 and 40 (*p* < 0.05). Accordingly, BW were significantly lower at the highest AME reduction of 75 kcal/kg compared to all other treatments on day 35 (*p* < 0.05). Also, on day 40, the lowest BW were reported for this treatment, but they only differed significantly from treatments with 25 kcal AME/kg reduction (*p* < 0.05).

Interactions between both dietary factors were found for days 7 and 21 (*p* < 0.05). On day 7, reported BW for DL-Met and MHA-FA-fed birds without AME reduction differed significantly (*p* < 0.05), whereas all remaining treatments did differ neither among each other nor from those without energy reduction. Regressing the BW differences between DL-Met- and MHA-FA-fed broilers against decreasing dietary AME revealed that BW converged by 1.8 g per 10 kcal AME/kg reduction at day 7 (Table 4; r^2^: 0.95) while it was 7 g at day 21 (r^2^: 84%). On day 21, BW of DL-Met-fed birds both without and with 25 kcal AME/kg reduction were significantly higher than those of the corresponding MHA-FA treatments (*p* < 0.05). As shown in Table 5, BWG per week were significantly higher in DL-Met-fed broilers in comparison to birds fed MHA-FA from days 1 to 7, 8 to 14, and 29 to 35 (*p* < 0.05). Similarly to BW, BWG were also significantly affected by dietary AME reduction during the periods 29–35 and 36–40 days of age. In both weeks, BWG were lowest with the strongest AME reduction (−75 kcal AME/kg; *p* < 0.05). Only for the first week was an interaction of dietary factors found with the highest BWG in Treatment 1 and lowest BWG in Treatment 2 (*p* < 0.05), whilst performance of all other treatments was intermediate. The interaction (*p* < 0.05) which was found for the period 1–7 days of age reflects the finding for the interactions of the 7 day BW. Regressing the differences between DL-Met and MHA-FA treatments against dietary AME reduction revealed that the difference decreased by 1.7 g/d (Table 5; r^2^: 0.96).

Although there were a few tendencies (0.50 < *p*< 0.10) reported for weeks 15–21, 22–28, and 36–40 days of age, the choice of methionine supplement affected FI significantly only during the first experimental week (*p* < 0.05, Table 6). In this case, FI was higher with DL-Met. The interaction reported for the period 15–21 days of age suggested a linear decrease in the difference between DL-Met and MHA-FA treatments along with dietary AME reduction converging by 5.8 g per 10 kcal/kg AME reduction (Table 6; r^2^: 65%).

The reed conversion ratio was hardly affected by dietary treatments (Table 7). However, birds fed DL-Met-supplemented diets had 0.05 kg/kg lower FCR from day 36 to 40, resulting in an overall (1–40 days) lower cumulated FCR by 2 points (*p* < 0.05). However, when FCR was corrected for mortality, this difference disappeared (*p* > 0.05). The dietary AME reduction with concurrent supplementation with GAA affected FCR especially from 29 to 40 days, which then also affected cumulated FCR from 1 to 35 days (*p* < 0.05). The treatments with 75 kcal AME/kg reduction showed the highest FCR during the week 29–35 days, whereas it was lowest in the following week (*p* < 0.05). Corrected FCR cumulated over the entire experimental period was significantly higher with 75 kcal AME/kg reduction compared to 25 kcal AME/kg reduction (*p* < 0.05). No interactions between dietary factors were found for FCR.

#### 3.3.2. Carcass Evaluation

Statistical analysis of carcass data revealed that methionine products did not have an impact on liveweight, breast meat, and thigh weight nor on yields (*p* > 0.05, Table 8). However, the choice of methionine source interacted with AME reduction and GAA supplementation. So, BW of broilers selected for carcass evaluation were highest in Treatment 1 and differed significantly from BW of broilers selected from Treatments 7 and 8 (*p* < 0.05). Moreover, BW of broilers of Treatment 8 were lowest and differed significantly from those of Treatments 1, 2, 5, and 9 (*p* < 0.05). The interaction for carcass weights was similar to that for liveweights. Regarding breast (*p* < 0.10) and legs (*p* < 0.05), the lowest weights were also found in broilers of Treatment 8, which differed significantly (in case of legs) from those reported for Treatments 1, 2, 4, and 9. These interactions were mainly determined by the significant direct effects of AME reduction. Particularly −75 and −50 kcal AME/kg showed the lowest liveweight, carcass weight, and leg meat weights compared to the standard treatments without AME reduction (*p* < 0.05).

## 4. Discussion

The study was conducted to evaluate interactions between the energy-sparing potential of GAA added to gradually energy-reduced diets and the choice of either DL-Met or the methionine precursor MHA-FA under commercial broiler feeding conditions. Indeed, the sparing potential of supplementation of 600 g/t GAA for AME has been reported previously [9,33,34,35,36,37,38,39] and based on such studies, a decision-support tool has been developed to guide nutritionists to adequately use 600 g/t GAA in poultry diets [40]. This tool suggests energy saving potential between zero and >150 kcal AME per kg of feed depending on the dietary settings of the unsupplemented feed. One example for a successful application of this tool has recently been reported by Lemme et al. [41]. Accordingly, dietary AME reductions by 51, 62, 72, and 99 kcal/t in starter, grower I, grower II, and finisher diets accompanied by 600 g/t GAA supplementation did not affect performance of broilers in a large-scale broiler operation but allowed for feed cost savings by up to 15 € per ton of feed. So, feed cost savings are targeted when applying the AME savings concept for GAA. In the current experiment AME was reduced by 25, 50, and 75 kcal/kg in all phases of the five-phase feeding program. Gradual energy reductions were achieved by partial replacement of corn with inert Kaolin (Supplementary Materials, Tables S1–S5). While energy reductions under many commercial conditions and, therefore, feed cost savings would be achieved especially by dietary fat reduction, there were no fat additions to standard diets (only 0.3% in grower feeds), which could have been reduced. Therefore, the experimental setup differed from the above-mentioned references on GAA in energy-reduced diets but indicates limitations for the implementation of the energy saving concept for GAA under commercial feed production conditions.

The second dietary factor in this experiment was the use of either DL-Met or MHA-FA to balance dietary sulfur amino acid levels. We hypothesized that an overestimation of the bioefficacy of MHA-FA might limit performance of broilers compared to DL-Met. An overestimation of the bioefficacy of MHA-FA may also limit the methylation of GAA into creatine due to insufficient availability of methyl groups. A number of simultaneous dose–response feeding trials with broilers using graded levels of either MHA-FA or DL-Met revealed an average bioefficacy of 63% for MHA-FA relative to DL-Met [23]. In fact, this means that any supplementation level of MHA-FA can be replaced by DL-Met additions being 63% as high as MHA-FA levels at the product level without compromising the growth, feed conversion, or meat yield of broilers [23]. Moreover, this paper provided evidence that the methodology to determine the bioefficacy is valid [23], and another comprehensive meta-analysis of feeding trials challenging the bioefficacy of 65% [24] clearly confirmed the applicability of the concept. While an overestimation of the bioefficacy of MHA-FA relative to DL-Met would result in performance depression at marginal or deficient dietary Met+Cys supply, it would not have an effect on performance when supplemented higher than Met+Cys recommendations [24]. In any case, there is a feed cost saving potential when using DL-Met at a bioefficacy of 65%, and the price ratio between products is >65% [23,24]. In the current trial, a scenario was mimicked which is sometimes found in commercial broiler production and, accordingly, a bioefficacy of 80% was assumed for MHA-FA. By assuming 80% bioefficacy, the targeted SID Met+Cys:SID Lys ratio for pre-starter, starter, grower, finisher I, and finisher II diets were 75, 75, 76, 76, and 76%, while assuming a bioefficacy of 65%, as done in the DL-Met65 treatment, would reveal ratios of 70, 70, 72, 72, and 72, respectively. The latter values would suggest marginal dietary Met+Cys supply according to breeder recommendation [31]. Indeed, BW of treatments with DL-Met compared to those of treatments with MHA-FA both in Part A (only two treatments, Table 1) and Part B (Table 5) were significantly higher, indicating that MHA-FA did not provide enough SID Met+Cys for optimizing growth. In Part A, BWG were not always significantly higher in DL-Met but sometimes numerically. Indeed, at days 28 and 35, these differences were less pronounced, which can be traced back to the low recovery of supplemental DL-Met in finisher I diets. On the other hand, the general effects on growth were not reflected in feed consumption and feed conversion ratios (Table 2 and Table 3) nor in carcass and meat yields (Table 4). Often, birds respond more sensitively in FCR or breast meat yield to marginal dietary Met+Cys supply [24,42,43] but not in the present case. Maynard et al. [44] compared three inclusion levels of MHA-FA and DL-Met, which were added in a corresponding product weight ratio of 100:88, in Cobb 500 broilers. The inclusion levels were below (63%), at (75%), and above (87%) optimal Met+Cys:Lys ratio in the 35-day trial. The low Met+Cys supply resulted in significantly lower final BW and higher FCR. While no statistically significant performance differences between the two products were detected nor interactions with dosage, it is remarkable that at a low (deficient) dietary Met+Cys:Lys ratio, the final BW of MHA-FA-fed broilers was 117 g/broiler lower than that of DL-Met-fed broilers, while this difference shrank to 31 g/broilers at the recommended Met+Cys supply [44]. The respective FCR were 4 and 2 higher in the MHA-FA treatments. These numbers, therefore, support the findings of the current experiment that a performance impact, and therefore the lower bioefficacy of MHA-FA, can only be demonstrated at marginal dietary Met+Cys supply while at or above the requirement, this effect is reduced or veiled.

Interestingly, the treatment where DL-Met was supplemented as high as 65% of the MHA-FA dose, which indicated that this dosage was sufficient to replace MHA-FA without compromising performance until day 35 (Table 2), as is in line with the outcome of a comprehensive meta-analysis [24]. Moreover, at termination of the experiment at day 40, the average BW of the DL-Met 65 treatment was even significantly higher than reported for the MHA-FA treatment, although MHA-FA-fed broilers were still 3.2% ahead of breeder recommendation [31]. While statistically not significant, this effect was present at day 35, which is remarkable insofar as the recovery of free methionine in finisher I diets suggested an unexpected low Met+Cys supply and a DLM:MHA-FA ratio of about 53%. These effects were reflected in FCR but disappeared with correction for mortality. Anyhow, such an advantage of DL-Met over MHA-FA is confirmed by another study performed with Cobb 500 broilers in Brazil. Pagliari Sangali et al. [45] found bioefficacies of 52 and 57% for MHA-FA for BWG and FCR. These values, which are lower than 65%, suggest a potential for outperforming MHA-FA-fed broilers even at an applied bioefficacy of 65%. More recently, a significantly higher breast meat yield was reported for DL-Met-fed compared to MHA-FA-fed Ross 708 broilers, whereas BWG and FCR were not different between products when supplemented in a 100 MHA-FA:65 DL-Met weight ratio [46].

Overall, it is concluded that an overestimation of the bioefficacy of MHA-FA relative to DL-Met bears the risk of limited broiler performance and, of course, disadvantages on profitability due to higher diet prices and feed cost. In fact, pre-starter, starter, grower I, grower II, and finisher diets using 80% as much MHA-FA as DL-Met were 0.22, 0.18, 0.16, 0.13, and 0.09 US$/t cheaper, while they were 1.32, 1.12, 1.05, 0.85, and 0.70 US$/t more expensive than DL-Met65 diets, respectively. This is interesting insofar as the results clearly confirm that the replacement of MHA-FA by DL-Met65 allows at least for similar, potentially even for better, performance with DL-Met.

The above discussion suggests that assuming 80% bioefficacy for MHA-FA relative to DL-Met is suboptimal and consequently, this might interact with the efficient use of GAA, which was evaluated in Part B of the study. However, we could not find consistent statistically significant interactions between methionine products and dietary energy reduction for the performance parameters considered. Interactions found for BW at day 7 and 21 as well as for BWG from 1 to 7 days of age and FI from 15 to 21 days of age indicated that the performance differences between the DL-Met and MHA-FA treatments reduced or converged with increasing dietary AME reduction. Numerically, the final BW and BWG of MHA-FA treatments, which were significantly lower than with DL-Met in the case of no AME reduction, appeared rather unaffected with decreasing dietary AME level whilst DL-Met treatments were affected in a linear way. The latter does not suggest that MHA-FA would better support the efficiency of supplemented GAA in energy-reduced diets but at best, suggests that DL-Met supplementation could not maintain the high efficiency achieved at standard AME. Interestingly, the rather low DLM supplementation in finisher I feeds did not negatively impact BWG or FCR, suggesting that even a DLM:MHA-FA ratio lower than 65% compromised performance. Thus, the observed deviation from target levels had no detrimental impact on broiler responses. To our knowledge, there is no publication available addressing interactions between dietary methionine products and GAA efficiency. Compared to MHA-FA, the use of DL-Met in broiler feed upregulated the creatine metabolism in breast muscle tissue while it strongly downregulated the mitochondrial uncoupling in breast muscle tissue particularly in young broilers [27]. These and many more effects of methionine supply and methionine products on metabolic pathways were described but the up- and downregulation of gene activities are not necessarily translated into biological responses. Indeed, broilers fed methionine-deficient diets grew significantly less, had significantly higher FCR, and yielded significantly less breast meat in the transcriptomics study by Klünemann et al. [27]. However, even though methionine products strongly affected genes of the energy metabolism in breast meat tissue in trials, broilers fed 65% as much DL-Met as MHA-FA had a similar final BW, FCR, and breast meat yield, indicating that such gene-related effects were not translated into performance effects [27]. The current experiment showed differences between DL-Met- and MHA-FA-fed broilers, but it is hard to speculate whether a more demanding energy metabolism with decreasing dietary AME supply redirects methionine to creatine formation, which consequently might limit methionine availability for growth.

Earlier studies on GAA supplementation suggested a rather efficient transformation of GAA into creatine, resulting in 15 to 20% higher creatine concentrations in broiler breast meat with 600 to 1200 g/t GAA supplementation [9]. Moreover, not only was creatine content in breast muscle increased, but phosphorylated creatine was also increased by GAA supplementation, resulting in an increased ratio of phosphorylated creatine to ATP [9,11,12,13,14]. It has been reported that at least 80% of the total energy is exported out of mitochondria in the form of phosphorylated creatine, which makes creatine a key constituent for energy metabolism [16]. However, in one reported example, GAA was inefficient when dietary Met+Cys was below requirement [30], whereas in another study, deficient and adequate dietary Met+Cys levels did not show any interaction with GAA effectiveness [47]. It is concluded that under normal commercial conditions aiming for satisfying nutrient requirements of broilers, a resulting marginal Met+Cys supply due to an overestimation of the bioefficacy of MHA-FA is unlikely to interact with the efficiency of supplemental GAA but rather results in reduced biological performance. The general absence of interactions (except for body weights at days 7 and 21; see above) might thus be a consequence of a physiological prioritization of the metabolism for using sulfur amino acids for different metabolic pathways. The methionine metabolism includes a multitude of biochemical pathways which are differently triggered, e.g., by dietary Met+Cys supply, as well as the choice of methionine sources or methionine precursors [27]. The comprehensive transcriptomics study revealed that a methionine deficiency up- or downregulates genes of metabolic pathways involved in amino acid metabolism, lipid metabolism, and energy metabolism but is also related to oxidative stress response, detoxification, or the immune system [27]. As described in the Introduction the entrance of homomocysteine into either the transulfuration or the remethylation pathway would be affected by dietary methionine supply [28]. Moreover, in breast meat tissues, an upregulation of mitochondrial fatty acid beta-oxidation and mitochondrial uncoupling due to dietary methionine deficiency was found [27]. This is interesting insofar as mitochondrial uncoupling means that the normal oxidative phosphorylation and ATP production in mitochondria is partially or fully disrupted. ATP is synthesized by oxidative phosphorylation in the mitochondria, and for transporting the energy-rich phosphoryl groups out of the mitochondria, creatine serves as a shuttle [15,22]. Therefore, the results obtained by Klünemann et al. [27] describe a link between methionine metabolism and GAA or creatine metabolism. With respect to the absence of overall consistent statistical interactions in the current experiment, it is assumed that sufficient methyl groups out of the homocysteine cycle were available to transform GAA into creatine at the expense of methionine availability to maximize body weight and body weight gain.

Statistically significant interactions were detected for liveweights of birds selected for carcass analysis as well as for respective carcass and leg weights (*p* < 0.05), indicating that, especially with DL-Met supplementation, weights would linearly decline with decreasing dietary energy (r^2^: 0.88; based on means). Thus, weights of cuts must be interpreted with caution because the body weights of these selected broilers did not represent the final weights of the growth study. So, on day 40, the body weights of MHA-FA-fed broilers were 81, 102, 56, and 10 g lower than DL-Met-fed broilers in treatments with no, 25 kcal/kg (+GAA), 50 kcal/kg (+GAA), and 75 kcal/kg reduced ME, respectively. In broilers selected for carcass analysis, the differences were 73, −46, 52, and −131 g, which means, especially at 25 and 75 kcal/kg energy reduction, selected MHA-FA-fed broiler were even heavier than DL-Met-fed broilers. Therefore, weights of cuts were not representative; however, with respect to yields (% of liveweight), there were no interactions at all. Accordingly, supplementation of GAA to gradually reduced dietary AME allowed for the same carcass yield, breast meat yield, and leg meat yield.

The experimental design included four dietary energy levels. However, when AME was reduced by 25, 50, and 75 kcal/kg, diets were supplemented with 600 g/t GAA. This was done to evaluate up to which AME reduction GAA supplementation can maintain broiler performance. Several publications that looked into this topic suggest a saving potential of 100 kcal/kg AME and more [9]. This sparing potential may depend on dietary settings, age of broilers, housing conditions, etc. Therefore, it is not surprising that some references suggested an AME-sparing potential of 50 kcal/kg [5,34,38,39,48], of 100 kcal/kg [33,36,49], 150 kcal/kg [50], or even 200 kcal/kg [6] for 600 g/t GAA. Others who tested even 150 and/or 300 kcal/kg AME reduction failed to maintain performance with 600 g/t GAA [35,37,51]. However, it should be noted that the energy reduction was always achieved by a reduction in dietary oil addition, which is in contrast to the current study where an inert filler was used to dilute the energy supply by corn. This was necessary because standard feeds (control) contained no or very little oil. The low oil inclusions in standard feeds even though being in line with breeder recommendation [31] were low. With using kaolin as an inert filler dietary AME could be reduced to even lower levels. In this context, it is remarkable that broiler performance could be maintained with GAA supplementation with 75 kcal AME/kg reduction.

The absence of significant effects for AME reduction may suggest that final BW and FCR as well as carcass, breast meat, and leg yield could basically be maintained with 600 g/t GAA up to 75 kcal AME/kg reduction. Numerically, performance suffered with 75 kcal AME/kg reduction. Therefore, we need to be cautious with concluding that GAA compensated more than 50 kcal AME/kg. However, a decline in growth and increase in FCR occurred only after day 21 and, thus, the results may suggest an AME-sparing potential of 75 kcal/kg at least during the pre-starter, starter, and grower phases. In contrast, the tool proposed by Dorigam et al. [40] would suggest lower AME-sparing potentials of 45, 57, and 70 kcal/kg for the pre-starter, starter, and grower phases, which then increase to 85 and 100 kcal/kg for the finisher I and finisher II phases. A 75 kcal/kg reduction plus GAA supplementation in the pre-starter, the starter, and the grower phases did not impair performance, indicating that there might be even higher potential for AME sparing. As a 75 kcal AME/kg reduction seemed not to work out in the current trial, it might be speculated whether the dilution with kaolin instead of reducing oil supplementation to the diets played a role. Actually, only during the phase of 20 to 35 days of age was FI 73 and 87 g lower in the DLM and MHA-FA treatments at −75 kcal AME/kg than the respective control treatments, while during all remaining phases, FI was hardly affected by the highest AME reduction. Consequently, the broilers of these treatments showed a reduced growth of −94 g on average. It is not likely that FI and BWG at lowest AME levels during this phase were affected by the low DLM supplementation because the MHA-FA treatment had the same magnitude of response.

## 5. Conclusions

The experimental design was split into two parts with the intention to give an example on the optimal exchange ratio of supplemental MHA-FA and DL-Met on the one hand. On the other hand, the second part of the study used an unfavourable replacement ratio of MHA-FA and DL-Met aiming to provoke an interaction between the effectiveness of GAA as an energy-sparing feed additive and the methyl group supply by either DL-Met or MHA-FA. From the first part of the study, it is clearly concluded that an overestimation of the bioefficacy of MHA-FA relative to DL-Met bears the risk of compromised broiler performance and, of course, related economic disadvantages due to higher diet prices and feed cost. A replacement of MHA-FA by DL-Met with an applied bioefficacy of 65% allowed at least for similar, potentially even for better, performance with DL-Met while there is potential for feed cost savings. The second part of the study did not reveal consistent findings on an interaction between MHA-FA and DL-Met and the effectiveness of GAA in gradually energy-reduced broiler feeds. Indeed, MHA-FA supplementation 80% as high as DL-Met resulted in significantly lower BW, suggesting that the requirement for sulfur amino acids to maximize performance was not achieved, but apparently the availability of methyl groups for the methylation process of GAA to creatine was not affected. Furthermore, the feeding trial indicated that an addition of 600 g/t GAA can compensate for up to a 75 kcal/kg reduction in AME in diets without compromising broiler performance.

## Figures and Tables

**Table 1:** Description of experimental treatments.

Treatment	Methionine Source	Dosage of Methionine Source ^2^	Reduction of Metabolizable Energy	Addition of Guanidinoacetic Acid Product ^3^
**PART A**				
1 ^1^	DL-Met		Standard	-
2 ^1^	MHA-FA	125% as much as Trt 1	Standard	-
3	DL-Met	65% as much as Trt 2	Standard	-
				
**PART B**				
1 ^1^	DL-Met		Standard	-
2 ^1^	MHA-FA	125% as much as Trt 1	Standard	-
4	DL-Met		25 kcal/kg	600 g/t
5	MHA-FA	125% as much as Trt 4	25 kcal/kg	600 g/t
6	DL-Met		50 kcal/kg	600 g/t
7	MHA-FA	125% as much as Trt 1	50 kcal/kg	600 g/t
8	DL-Met		75 kcal/kg	600 g/t
9	MHA-FA	125% as much as Trt 1	75 kcal/kg	600 g/t

^1^ Treatments 1 and 2 were the same for PART A and PART B. ^2^ Relative dosages refer to product weights; a methionine activity of 80% was assumed for MHA-FA, resulting in a 25% higher inclusion level than DL-Met supplementation. ^3^ GuanAMINO^®^ containing minimum of 96% or 576 g/kg guanidinoacetic acid.

**Table 2:** Final body weights and mortality (top), feed consumption (middle), and feed conversion ratio (bottom) of broilers fed diets supplemented with DL-Met at two concentrations and MHA-FA (Part A).

	Final Body Weight (g)												
Trt	Met Source	Day of Age											Mortality (%)
		1	7	14		21		28		35		40	
**1**	**DL-Met**	43.4 ±0.8	177 ±4.2 ^a^	504 ±8.1 ^a^		1130 ±15.3 ^a^		1903 ±24.6		2828 ±34.1		3462 ±36 ^a^	6.00 ±2.030
**2**	**MHA-FA 88%**	43.2 ±0.11	164 ±3.5 ^b^	477 ±7.5 ^b^		1081 ±22.4 ^b^		1869 ±21.1		2766 ±36.9		3381 ±35 ^b^	3.69 ±1.046
**3**	**DL-Met65**	43.4 ±0.11	171 ±5.9 ^ab^	489 ±11.7 ^ab^		1102 ±19.4 ^ab^		1899 ±29.9		2840 ±35.5		3464 ±40 ^a^	6.46 ±2.542
	*p* values	0.124	0.04	0.02		0.05		0.33		0.09		0.04	0.31
									
	**Feed intake (g)**												
	**Met source**	1–7	8–14		15–21		22–28		29–35		36–40		1–40
**1**	**DL-Met**	128 ±5.5	412 ±7.1		823 ±17.8		1169 ±18.3		1121 ±29.9		1101 ±20.0		4719 ±39.8
**2**	**MHA-FA 88%**	118 ±3.2	407 ±11.0		795 ±14.1		1162 ±17.2		1137 ±36.0		1110 ±15.3		4727 ±75.7
**3**	**DL-Met65**	120 ±7.6	411 ±12.1		817 ±12.1		1175 ±23.1		1113 ±31.2		1130 ±18.8		4765 ±66.8
	*p* values	0.21	0.89		0.14		0.80		0.74		0.25		0.74
	**Feed conversion (g/g)**												
	**Met source**	1–7	1–14		1–21		1–28		1–35		1–40		1–40 corrected
**1**	**DL-Met**	0.969 ±0.0076	1.170 ±0.0091		1.269 ±0.0052		1.362 ±0.0042		1.312 ±0.0138		1.390 ±0.0104 ^a^		1.434 ±0.0158
**2**	**MHA-FA 88%**	0.953 ±0.0249	1.203 ±0.0216		1.270 ±0.0154		1.358 ±0.0089		1.328 ±0.0166		1.421 ±0.0068 ^b^		1.433 ±0.0120
**3**	**DL-Met65**	0.934 ±0.0207	1.188 ±0.0215		1.272 ±0.0099		1.359 ±0.0098		1.300 ±0.0189		1.388 ±0.0062 ^a^		1.431 ±0.0144
	*p* values	0.221	0.21		0.95		0.91		0.22		<0.01		0.97

^a,b^ Different superscripts within columns and performance criterion indicate significant performance differences (*p* < 0.05, Tukey test).

**Table 3:** Body weights and weights of carcass, breast meat, and thighs as well as carcass, breast meat, and thigh yield of broilers selected for carcass analysis and fed diets supplemented with DL-Met at two concentrations and MHA-FA (Part A).

		Absolute Weight (g)				Carcass and Parts Yield (% of Liveweight)		
Trt		Body Weight	Carcass Weight	Breast Meat	Thighs	Carcass	Breast Meat	Thighs
**1**	**DL-Met**	3380 ±46.4 ^a^	2421 ±44.4 ^a^	1041 ±33.4 ^a^	757 ±17.9	71.58 ±0.6436	30.80 ±0.8000	22.42 ±0.4474
**2**	**MHA-FA 88%**	3303 ±57.5 ^b^	2371 ±47.4 ^ab^	1004 ±33.3 ^ab^	757 17.8	71.78 ±0.5995	30.39 ±0.7525	22.91 ±0.4047
**3**	**DL-Met65**	3256 ±46.8 ^b^	2341 ±43.9 ^b^	990 ±32.3 ^b^	739 ±16.2	71.86 ±0.6902	30.39 ±0.8277	22.69 ±0.4213
	*p* values	<0.01	<0.01	0.03	0.10	0.74	0.59	0.14

^a,b^ Different superscripts within columns indicate significant performance differences (*p* < 0.05, Tukey test).

**Table 4:** Body weights of broilers fed diets with different sources and concentrations of methionine products and decreasing dietary energy combined with 600 g/t supplemental guanidinoacetic acid in consecutive feeding phases and cumulated overall (Part B).

Trt	Met Source	AME Reduction	D1	D7	D14	D21	D28	D35	D40	Mortality
1	DL-Met	0	43.4 ±0.8	177 ±4.3 ^a^	504 ±8.1	1130 ±15.3 ^a^	1903 ±24.6	2828 ±34.1	3462 ±36.3	6.00 ±2.030
2	MHA-FA	0	43.2 ±0.11	164 ±3.5 ^b^	477 ±7.5	1081 ±22.4 ^b^	1869 ±21.1	2766 ±36.9	3381 ±35.0	3.69 ±1.046
4	DL-Met + 600 g/t GAA	−25 kcal	43.2 ±0.10	176 ±2.5 ^ab^	503 ±5.8	1127 ±11.2 ^a^	1922 ±22.8	2872 ±36.1	3504 ±44.4	5.23 ±2.249
5	MHA-FA + 600 g/t GAA	−25 kcal	43.2 ±0.10	166 ±4.6 ^ab^	484 ±10.4	1082 ±23.3 ^b^	1862 ±25.3	2774 ±36.3	3402 ±40.8	6.46 ±2.059
6	DL-Met + 600 g/t GAA	−50 kcal	43.2 ±0.10	171 ±4.7 ^ab^	496 ±9.8	1108 ±24.8 ^ab^	1889 ±31.0	2802 ±38.7	3442 ±44.3	7.69 ±2.941
7	MHA-FA + 600 g/t GAA	−50 kcal	43.2 ±0.12	169 ±4.3 ^ab^	492 ±10.5	1103 ±20.8 ^ab^	1878 ±23.8	2750 ±45.0	3386 ±35.0	5.23 ±1.930
8	DL-Met + 600 g/t GAA	−75 kcal	43.2 ±0.11	169 ±4.5 ^ab^	500 ±8.5	1096 ±17.2 ^ab^	1875 ±25.2	2691 ±42.0	3352 ±47.9	6.15 ±1.580
9	MHA-FA + 600 g/t GAA	−75 kcal	43.2 ±0.10	169 ±3.3 ^ab^	491 ±6.8	1092 ±14.1 ^ab^	1861 ±33.9	2679 ±43.6	3342 ±51.0	5.54 ±2.265
**Factorial analysis**										
	DL-Met		43.3 ±0.10	173 ±4.1 ^a^	501 ±8.0 ^a^	1115 ±18.2 ^a^	1897 ±26.3 ^a^	2798 ±46.1 ^a^	3440 ±48.2 ^a^	6.27 ±2.221
	MHA-FA		43.2 ±0.11	167 ±3.9 ^b^	486 ±9.0 ^b^	1090 ±20.2 ^b^	1867 ±25.8 ^b^	2743 42.3 ^b^	3378 ±40.8 ^b^	5.23 ±1.873
		0	43.3 ±0.10	170 ±4.6	490 ±9.5	1104 ±21.5	1885 ±23.5	2796 ±37.1 ^a^	3420 ±38.8 ^ab^	4.85 ±1.633
		−25 kcal	43.3 ±0.10	170 ±4.6	490 ±9.5	1104 ±21.5	1885 ±23.5	2796 ±37.1 ^a^	3420 ±38.8 ^ab^	4.85 ±1.633
		−50 kcal	43.2 ±0.10	171 ±4.1	494 ±9.1	1104 ±20.2	1892 ±26.7	2823 ±41.0 ^a^	3453 ±46.9 ^a^	5.85 ±2.128
		−75 kcal	43.2 ±0.11	170 ±4.4	494 ±10.0	1106 ±22.5	1883 ±27.1	2776 ±42.5 ^a^	3414 ±40.8 ^ab^	6.46 ±2.491
***p*-values**										
	Met source		0.21	**<0.01**	**<0.01**	**<0.01**	**0.02**	**<0.01**	**<0.01**	0.30
AME reduction			0.42	0.59	0.43	0.47	0.27	**<0.01**	**<0.01**	0.42
Met sources × AME reduction ^1^			0.69	**0.01**	0.06	**0.04**	0.31	0.24	0.15	0.88

^a,b^ Different superscripts within columns and performance criteria indicate significant performance differences (*p* < 0.05, Tukey test). ^1^ Interactions for days 7 and 21 were evaluated by linear regression by plotting the average treatment differences between DL-Met and MHA-FA treatments against dietary AME reduction: BW-difference day 7 = −13.3–0.19 × AME reduction, kcal/kg, r^2^: 0.95; BW-difference day 21 = −52.0–0.70 × AME reduction, kcal/kg, r^2^: 0.84.

**Table 5:** Body weight gain (BWG) of broilers fed diets with different sources and concentrations of methionine products and decreasing dietary energy combined with 600 g/t supplemental guanidinoacetic acid in consecutive feeding phases and cumulated overall (Part B).

Trt	Met Source	AME Reduction	Days 1–7 ^1^	Days 8–14	Days 15–21	Days 22–28	Days 29–35	Days 36–40
1	DL-Met	0	133 ±4.2 ^a^	327 ±4.7	613 ±16.9	786 ±12.0	925 ±24.4	634 ±21.5
2	MHA-FA	0	121 ±3.5 ^b^	313 ±4.3	604 ±18.2	787 ±15.4	898 ±25.7	614 ±23.5
4	DL-Met + 600 g/t GAA	−25 kcal	132 ±2.4 ^ab^	327 ±3.8	624 ±7.4	795 ±18.9	951 ±17.6	632 ±19.1
5	MHA-FA + 600 g/t GAA	−25 kcal	123 ±4.6 ^ab^	318 ±6.3	598 ±14.7	780 ±16.6	912 ±32.0	628 ±16.7
6	DL-Met + 600 g/t GAA	−50 kcal	128 ±4.7 ^ab^	325 ±5.6	612 ±17.2	781 ±13.3	913 ±17.4	640 ±27.9
7	MHA-FA + 600 g/t GAA	−50 kcal	126 ±4.3 ^ab^	323 ±6.3	611 ±12.6	775 ±13.0	873 ±28.6	636 ±26.3
8	DL-Met + 600 g/t GAA	−75 kcal	126 ±4.5 ^ab^	330 ±4.3	597 ±12.9	778 ±13.1	816 ±29.5	661 ±27.7
9	MHA-FA + 600 g/t GAA	−75 kcal	126 ±3.3 ^ab^	322 ±4.6	602 ±8.5	769 ±27.4	818 ±23.9	662 ±27.4
	**Factorial analysis**							
	DL-Met		130 ±4.1 ^a^	328 ±4.6 ^a^	612 ±14.2	785 ±14.4	901 ±30.6 ^a^	642 ±24.1
	MHA-FA		124 ±3.9 ^b^	319 ±5.5 ^b^	604 ±13.7	778 ±18.6	875 ±30.7 ^b^	635 ±24.2
		0	127 ±4.6	320 ±5.3	609 ±17.4	787 ±13.6	911 ±25.2 ^a^	624 ±22.5 ^b^
		−25 kcal	128 ±4.1	323 ±5.4	611 ±12.7	788 ±17.7	931 ±26.5 ^a^	630 ±17.6 ^ab^
		−50 kcal	127 ±4.4	324 ±5.8	612 ±14.8	778 ±13.0	893 ±24.7 ^a^	638 ±26.6 ^ab^
		−75 kcal	126 ±3.9	326 ±4.8	599 ±10.8	774 ±21.1	817 ±26.3 ^b^	662 ±27.0 ^a^
	***p*-Values**							
	Met-source		**0.01**	0.27	0.24	0.38	**0.05**	0.5
	AME reduction		0.60	0.08	0.35	0.18	**<0.01**	**0.02**
	Met-source × AME reduction		**0.01**	0.27	0.26	0.76	0.43	0.53

^a,b^ Different superscripts within columns indicate significant performance differences (*p* < 0.05, Tukey test). ^1^ The interaction for the period 1–7 days of age was evaluated by linear regression by plotting the average treatment differences between DL-Met and MHA-FA treatments against dietary AME reduction: BWG-difference 1–7 days = −12.2–0.17 × AME reduction, kcal/kg, r^2^: 0.96.

**Table 6:** Feed consumption (FC) of broilers fed diets with different sources and concentrations of methionine products and decreasing dietary energy combined with 600 g/t supplemental guanidinoacetic acid in consecutive feeding phases and cumulated overall (Part B).

Trt	Met Source	AME Reduction	Days 1–7	Days 8–14	Days 15–21 ^1^	Days 22–28	Days 29–35	Days 36–40	Days 1–40
1	DL-Met	0	128 ±5.5	412 ±7.1	823 ±17.8 ^ab^	1169 ±18.3	1121 ±29.9	1101 ±20.0	4719 ±39.8
2	MHA-FA	0	118 ±3.2	407 ±11.0	795 ±14.1 ^b^	1162 ±17.2	1137 ±36.0	1110 ±15.3	4727 ±75.7
4	DL-Met + 600 g/t GAA	−25 kcal	128 ±3.2	415 ±7.0	839 ±16.3 ^a^	1162 ±17.0	1117 ±25.8	1107 ±15.9	4768 ±58.2
5	MHA-FA + 600 g/t GAA	−25 kcal	117 ±4.9	402 ±7.5	799 ±17.0 ^ab^	1139 ±13.9	1072 ±26.1	1132 ±14.8	4661 ±47.3
6	DL-Met + 600 g/t GAA	−50 kcal	119 ±7.5	412 ±9.2	816 ±20.3 ^ab^	1165 ±17.7	1082 ±34.2	1100 ±20.9	4694 ±74.2
7	MHA-FA + 600 g/t GAA	−50 kcal	116 ±6.5	412 ±10.4	821 ±15.3 ^ab^	1146 ±15.6	1081 ±30.7	1131 ±19.7	4706 ±67.9
8	DL-Met + 600 g/t GAA	−75 kcal	123 ±7.0	419 ±6.5	805 ±14.6 ^ab^	1165 ±12.8	1048 ±30.5	1114 ±23.7	4675 ±65.7
9	MHA-FA + 600 g/t GAA	−75 kcal	120 ±4.5	410 ±6.2	810 ±11.2 ^ab^	1155 ±17.6	1050 ±31.3	1118 ±20.3	4663 ±62.0
	**Factorial analysis**								
	DL-Met		125 ±6.1 ^a^	414 ±7.4	821 ±17.6	1165 ±16.1	1091 ±31.8	1106 ±19.9	4714 ±61.4
	MHA-FA		118 ±4.9 ^b^	408 ±8.9	807 ±14.7	1151 ±16.1	1085 ±33.1	1123 ±17.6	4689 ±63.3
		0	123 ±4.8	409 ±9.2	809 ±16.7	1166 ±17.4	1129 ±32.7 ^a^	1105 ±17.4	4723 ±60.7
		−25 kcal	122 ±4.7	408 ±7.6	819 ±18.3	1150 ±16.0	1094 ±27.1 ^ab^	1120 ±16.0	4714 ±56.5
		−50 kcal	117 ±6.9	412 ±9.6	819 ±17.7	1156 ±16.8	1081 ±31.8 ^ab^	1115 ±20.9	4700 ±69.7
		−75 kcal	122 ±5.8	415 ±6.5	808 ±12.7	1160 ±15.2	1049 ±30.3 ^b^	1116 ±21.6	4669 ±62.6
	***p*-Values**								
	Met-source		**0.01**	0.11	0.07	0.06	0.65	0.07	0.42
	AME reduction		0.46	0.27	0.87	0.74	**<0.01**	0.49	0.19
	Met-source *×* AME reduction		0.26	0.99	**0.04**	0.94	0.99	0.89	0.79

^a,b^ Different superscripts within columns indicate significant performance differences (*p* < 0.05, Tukey test). ^1^ Interactions for the period 15–21 days of age was evaluated by linear regression by plotting the average treatment differences between DL-Met and MHA-FA treatments against dietary AME reduction: FC-difference days 15–21 = −36.1–0.58 × AME reduction, kcal/kg, r^2^: 0.65.

**Table 7:** Feed conversion ratio of broilers fed diets with different sources and concentrations of methionine products and decreasing dietary energy combined with 600 g/t supplemental guanidinoacetic acid in consecutive feeding phases and cumulated overall (Part B).

Trt	Met Source	AME Reduction	Days 1–7	Days 8–14	Days 15–21	Days 22–28	Days 29–35	Days 36–40	Days 1–40
1	DL-Met	0	0.969 ±0.0076	1.170 ±0.0091	1.269 ±0.0052	1.362 ±0.0042	1.312 ±0.0138	1.390 ±0.0104	1.434 ±0.0158
2	MHA-FA	0	0.953 ±0.0249	1.203 ±0.0216	1.270 ±0.0154	1.358 ±0.0089	1.328 ±0.0166	1.421 ±0.0068	1.433 ±0.0120
4	DL-Met + 600 g/t GAA	−25 kcal	0.966 ±0.0120	1.180 ±0.0076	1.265 ±0.0049	1.354 ±0.0070	1.294 ±0.0153	1.378 ±0.0163	1.409 ±0.0134
5	MHA-FA + 600 g/t GAA	−25 kcal	0.952 ±0.0132	1.177 ±0.0076	1.270 ±0.0069	1.351 ±0.0081	1.293 ±0.0139	1.388 ±0.0125	1.425 ±0.0112
6	DL-Met + 600 g/t GAA	−50 kcal	0.943 ±0.0200	1.171 ±0.0163	1.259 ±0.0066	1.361 ±0.0084	1.303 ±0.0106	1.381 ±0.0137	1.441 ±0.0126
7	MHA-FA + 600 g/t GAA	−50 kcal	0.912 ±0.0352	1.175 ±0.0117	1.273 ±0.0083	1.360 ±0.0083	1.321 ±0.0156	1.408 ±0.0135	1.437 ±0.0113
8	DL-Met + 600 g/t GAA	−75 kcal	0.958 ±0.0204	1.188 ±0.0159	1.280 ±0.0100	1.372 ±0.0060	1.346 ±0.0142	1.414 ±0.0135	1.451 ±0.0112
9	MHA-FA + 600 g/t GAA	−75 kcal	0.956 ±0.0162	1.185 ±0.0072	1.278 ±0.0028	1.361 ±0.0061	1.345 ±0.0142	1.414 ±0.0145	1.449 ±0.0050
	**Factorial analysis**								
	DL-Met		0.959 ±0.0160	1.178 ±0.0129	1.268 ±0.0075	1.362 ±0.0070	1.314 ±0.0154	1.391 ±0.0145 ^a^	1.434 ±0.0168
	MHA-FA		0.944 ±0.0242	1.185 ±0.0137	1.273 ±0.0093	1.358 ±0.0078	1.322 ±0.0166	1.408 ±0.0129 ^b^	1.436 ±0.0106
		0	0.961 ±0.0189	1.187 ±0.0178	1.269 ±0.0114	1.360 ±0.0069	1.320 ±0.0154 ^b^	1.406 ±0.0107	1.433 ±0.0136 ^ab^
		−25 kcal	0.959 ±0.0127	1.178 ±0.0075	1.267 ±0.0060	1.353 ±0.0074	1.294 ±0.0143 ^c^	1.383 ±0.0144	1.417 ±0.0125 ^a^
		−50 kcal	0.929 ±0.0288	1.173 ±0.0139	1.267 ±0.0079	1.360 ±0.0081	1.312 ±0.0136 ^ab^	1.395 ±0.0144	1.439 ±0.0169 ^ab^
		−75 kcal	0.957 ±0.0179	1.187 ±0.0121	1.279 ±0.0072	1.367 ±0.0064	1.345 ±0.0139 ^b^	1.414 ±0.0137	1.450 ±0.0085 ^b^
	***p*-Values**								
	Met-source		0.16	0.25	0.32	0.20	0.28	**0.01**	0.70
	AME reduction		0.36	0.84	0.14	0.08	**<0.01**	0.19	**0.02**
	Met-source × AME reduction		0.77	0.08	0.96	0.48	0.62	0.22	0.64

^a–c^ Different superscripts within columns and performance criteria indicate significant performance differences (*p* < 0.05, Tukey test).

**Table 8:** Carcass analysis of broilers fed diets with different sources and concentrations of methionine products and decreasing dietary energy combined with 600 g/t supplemental guanidinoacetic acid in consecutive feeding phases and cumulated overall (Part B).

Trt	Met Source	AME Reduction	Liveweight ^1^ g	Carcass Weight g	Breast g	Leg g	Carcass Yield % of LW	Breast Yield % of LW	Legs % of LW
1	DL-Met	0	3380 ±46.4 ^a^	2421 ±44.4 ^a^	1041 ±33.4	757 ±17.9 ^a^	71.58 ±0.644	30.80 ±0.800	22.42 ±0.447
2	MHA-FA	0	3303 ±57.5 ^ab^	2371 ±47.4 ^abc^	1004 ±33.3	757 ±17.8 ^a^	71.78 ±0.599	30.39 ±0.753	22.91 ±0.405
4	DL-Met + 600 g/t GAA	−25 kcal	3284 ±67.8 ^abc^	2391 ±55.3 ^ab^	1019 ±36.5	758 ±18.7 ^a^	72.81 ±0.516	31.00 ±0.737	23.08 ±0.424
5	MHA-FA + 600 g/t GAA	−25 kcal	3330 ±56.2 ^ab^	2397 ±47.2 ^ab^	1042 ±31.0	742 ±16.0 ^ab^	72.02 ±0.613	31.32 ±0.703	22.29 ±0.389
6	DL-Met + 600 g/t GAA	−50 kcal	3289 ±58.3 ^abc^	2362 ±53.0 ^abc^	1009 ±36.7	740 ±18.7 ^ab^	71.85 ±0.675	30.67 ±0.709	22.52 ±0.522
7	MHA-FA + 600 g/t GAA	−50 kcal	3237 ±71.8 ^bc^	2324 ±58.8 ^bc^	1005 ±73.3	739 ±20.2 ^ab^	71.84 ±0.927	31.05 ±2.147	22.82 ±0.380
8	DL-Met + 600 g/t GAA	−75 kcal	3201 ±60.2 ^c^	2293 ±52.6 ^c^	982 ±39.6	731 ±16.8 ^b^	71.63 ±0.573	30.65 ±0.887	22.84 ±0.414
9	MHA-FA + 600 g/t GAA	−75 kcal	3332 ±53.6 ^ab^	2392 ±53.6 ^ab^	1025 ±37.4	756 ±18.2 ^a^	71.73 ±0.710	30.70 ±0.895	22.69 ±0.487
	**Factorial analysis**								
		DL-Met	3287 ±63.8	2365 ±54.6	1012 ±37.3	746 ±18.4	71.98 ±0.631	30.78 ±0.781	22.82 ±0.781
		MHA-FA	3300 ±61.7	2371 ±52.7	1019 ±47.0	748 ±18.3	71.84 ±0.718	30.86 ±1.273	22.68 ±1.273
		0	3340 ±54.4 ^a^	2395 ±46.8 ^a^	1021 ±33.9	757 ±17.7 ^a^	71.69 ±0.618	30.58 ±0.774	22.88 ±0.434
		−25 kcal	3307 ±62.7 ^ab^	2394 ±51.1 ^a^	1031 ±33.9	749 ±17.5 ^ab^	72.42 ±0.585	31.16 ±0.718	22.68 ±0.435
		−50 kcal	3262 ±66.0 ^b^	2343 ±56.1 ^b^	1007 ±57.6	739 ±19.3 ^b^	71.85 ±0.805	30.86 ±1.599	22.67 ±0.457
		−75 kcal	3265 ±62.8 ^b^	2342 ±56.5 ^b^	1004 ±39.2	744 ±18.2 ^ab^	71.68 ±0.639	30.67 ±0.885	22.77 ±0.450
***p*-Values**									
Met-source			0.42	0.68	0.53	0.64	0.48	0.77	0.72
AME reduction			**<0.01**	**<0.01**	0.15	**0.03**	0.46	0.97	0.67
Met-source × AME reduction			**<0.01**	**<0.01**	0.06	**0.04**	0.69	0.57	0.49

^a–c^ Different superscripts within columns indicate significant performance differences (*p* < 0.05, Tukey test). ^1^ Liveweight response of DL-Met-fed broilers selected for carcass evaluation was evaluated by linear regression by plotting the average treatment means against dietary AME reduction: liveweight (DL-Met) = 3368 + 2.1 × AME reduction, kcal/kg, r^2^: 0.88.

## Data Availability

The raw data supporting the conclusions of this article will be made available by the authors on request.

## Supplementary Materials

The following supporting information can be downloaded at: https://www.mdpi.com/article/10.3390/ani16121811/s1, Table S1. Formulation and nutrient composition of pre-starter diet (days 0–7). Table S2. Formulation and nutrient composition starter diet (days 8–14). Table S3. Formulation and nutrient composition of grower diet (days 15–24). Table S4. Formulation and nutrient composition of finisher I diet (days 25–35). Table S5. Formulation and nutrient composition of finisher II diet (days 36–40). Table S6. Analyzed nutrient compositions of pre-starter diet (days 0–7). Table S7. Analyzed nutrient compositions of starter diet (days 8–14). Table S8. Analyzed nutrient compositions of grower diet (days 15–24). Table S9. Analyzed nutrient compositions of finisher I diet (days 25–35). Table S10. Analyzed nutrient compositions of finisher II diet (days 36–40).

## Abbreviations

The following abbreviations are used in this manuscript:

ATP	Adenosine Triphosphate
BW	Body Weight
BWG	Body Weight Gain
CP	Crude Protein
DM	Dry Matter
FCR	Feed Conversion Ratio
FI	Feed Intake
GAA	Guanidinoacetic Acid
ME	Metabolizable Energy
RE	Reduced Energy
SE	Standard Energy
TFC	Total Feed Consumed
