# Supplementary material for: Effects of DL-Methionine or Methionine Hydroxy Analogue-Free Acid Supplementation on Growth Performance and Carcass Yield of Broilers Fed Reduced Energy Diets with Guanidinoacetic Acid Supplementation
Source: Animals (Basel). 2026 Jun 11;16(12):1811. doi: 10.3390/ani16121811 (PMC13295774; doi:10.3390/ani16121811)
Supplement: Supplementary file 1 [file animals-16-01811-s001.zip › animals-4297979-supplementary.pdf]

Table S1. Formulation and nutrient composition of pre-starter diet (days 0–7).

	**Standard**			**−25 kcal/kg**		**−50 kcal/kg**		**−75 kcal/kg**	
	**DLM**	**MHA**	**DL65**	**DLM**	**MHA**	**DLM**	**MHA**	**DLM**	**MHA**
**Ingredient, %**									
Corn	578.76	577.93	577.93	569.17	569.33	561.16	560.32	553.16	551.31
Soybean meal (46% CP)	355.00	355.00	356.52	356.00	356.00	357.00	358.00	358.00	359.00
Meat & bone meal	25.00	25.00	25.00	26.00	26.00	26.00	26.00	26.00	26.00
Limestone	5.00	5.00	5.00	5.00	5.00	5.00	5.00	5.00	5.00
Dicalcium phosphate	10.00	10.00	10.00	10.00	10.00	10.00	10.00	10.00	10.00
Minerals Zn Se Cr	0.70	0.70	0.70	0.70	0.70	0.70	0.70	0.70	0.70
Sodium bicarbonate	2.98	2.98	2.98	2.97	2.97	2.96	2.96	2.95	2.95
Salt	3.07	3.07	3.07	3.07	3.07	3.08	3.08	3.08	3.08
Vitamin premix [1]	0.96	0.96	0.96	0.96	0.96	0.96	0.96	0.96	0.96
Mineral premix [2]	1.20	1.20	1.20	1.20	1.20	1.20	1.20	1.20	1.20
Enzyme starter	0.40	0.40	0.40	0.40	0.40	0.40	0.40	0.40	0.40
Choline chloride (75%)	0.57	0.57	0.57	0.57	0.57	0.57	0.57	0.57	0.57
L-Lysine sulphate 80	2.96	2.96	2.96	2.94	2.94	2.92	2.92	2.91	2.91
GAA	-	-	-	0.60	0.60	0.60	0.60	0.60	0.60
L-Valine	0.50	0.50	0.50	0.51	0.51	0.51	0.51	0.52	0.52
L-Threonine	1.57	1.57	1.57	1.57	1.57	1.58	1.58	1.58	1.58
MHA-FA	-	4.34	-		4.36	-	4.38	-	4.40
DL-Methionine	3.51	-	2.82	3.52	-	3.54	-	3.55	-
Narasin/Nicarbazin	0.63	0.63	0.63	0.63	0.63	0.63	0.63	0.63	0.63
Mycotoxin adsorbent	1.00	1.00	1.00	1.00	1.00	1.00	1.00	1.00	1.00
Formaldehyde	1.50	1.50	1.50	1.50	1.50	1.50	1.50	1.50	1.50
Organic acid	0.30	0.30	0.30	0.30	0.30	0.30	0.30	0.30	0.30
Probiotic	0.40	0.40	0.40	0.40	0.40	0.40	0.40	0.40	0.40
Filler	4.00	4.00	4.00	11.00	10.00	18.00	17.00	25.00	25.00
Total	1.000	1.000	1.000	1.000	1.000	1.000	1.000	1.000	1.000
**Calculated value, %**									
Crude protein	23.65	23.66	23.66	23.80	23.83	23.79	23.85	23.78	23.84
AMEn, kcal/kg	2.951	2.950	2.950	2.923	2.925	2.899	2.900	2.875	2.872
Fat	3.27	3.27	3.27	3.24	3.25	3.22	3.21	3.19	3.18

Crude fibre	2.23	2.23	2.23	2.22	2.22	2.21	2.21	2.20	2.20
Calcium	0.96	0.96	0.96	0.97	0.97	0.97	0.97	0.97	0.97
Available phosphorus	0.60	0.60	0.60	0.60	0.60	0.60	0.60	0.60	0.60
Dig Lys, %	1.30	1.30	1.30	1.30	1.30	1.30	1.30	1.30	1.30
Dig Met, %	0.66	0.66	0.66	0.66	0.66	0.67	0.67	0.67	0.67
Dig Met+Cys, %	0.98	0.97	0.97	0.97	0.98	0.98	0.98	0.98	0.98
Dig Thr, %	0.88	0.88	0.88	0.88	0.88	0.88	0.89	0.88	0.89
Dig Try, %	0.24	0.24	0.24	0.24	0.24	0.24	0.24	0.24	0.24
Dig Arg, %	1.40	1.40	1.40	1.44	1.44	1.45	1.45	1.45	1.45
Dig Iso, %	0.84	0.84	0.84	0.84	0.85	0.84	0.85	0.84	0.85
Dig Leu, %	1.61	1.61	1.61	1.61	1.61	1.61	1.61	1.60	1.61
Dig Val, %	0.99	0.99	0.99	0.99	0.99	0.99	0.99	0.99	0.99
Dig His, %	0.54	0.54	0.54	0.54	0.54	0.54	0.54	0.54	0.54
Dig Fen, %	0.94	0.94	0.94	0.94	0.94	0.94	0.94	0.94	0.94
Dig Gly, %	0.86	0.86	0.86	0.86	0.86	0.86	0.87	0.86	0.86
Met/Lys ratio	0.51	0.51	0.51	0.51	0.51	0.51	0.51	0.51	0.51
M+C/Lys ratio	0.75	0.75	0.75	0.75	0.75	0.75	0.75	0.75	0.75
Thr/Lys ratio	0.68	0.68	0.68	0.68	0.68	0.68	0.68	0.68	0.68
Try/Lys ratio	0.19	0.19	0.19	0.19	0.19	0.19	0.19	0.19	0.19
Arg/Lys ratio	1.08	1.08	1.08	1.11	1.11	1.11	1.11	1.11	1.11
Iso/Lys ratio	0.65	0.65	0.65	0.65	0.65	0.65	0.65	0.65	0.65
Leu/Lys ratio	1.24	1.24	1.24	1.24	1.24	1.24	1.24	1.23	1.23
Val/Lys ratio	0.76	0.76	0.76	0.76	0.76	0.76	0.76	0.76	0.76

Each ton of starter feed contains: [1] 12,000 KIU of vitamin AD3, 5160 KIU of vitamin D3, 84,000 IU of vitamin E, 4320 mg of vitamin K3, 3480 mg of vitamin B1, 8280 mg of vitamin B2, 4800 mg of vitamin B6, 36 mg of vitamin B12, 17,400 mg of pantothenic acid, 72,000 mg of niacin, 2280 mg of folic acid, 324 mg of biotin, and 132 g of BHT; [2] 1200 mg of cobalt (as cobalt sulfate), 18,000 mg of copper (as copper sulfate), 3600 mg of iodine (as calcium iodate monohydrate), 54,000 mg of iron (as ferrous sulfate), 120,000 mg of manganese (as manganese sulfate), 240 mg of selenium (as sodium selenite), and 96,000 mg of zinc (as zinc sulfate).

Table S2. Formulation and nutrient composition starter diet (days 8–14).

	Standard			−25 kcal/kg		−50 kcal/kg		−75 kcal/kg	
	**DLM**	**MHA**	**DL65**	**DLM**	**MHA**	**DLM**	**MHA**	**DLM**	**MHA**
**Ingredient, %**									
Corn	618.15	618.43	618.43	610.54	609.81	601.55	601.81	593.52	593.79
Soybean meal (46% CP)	310.00	310.00	313.37	311.00	311.00	312.00	312.00	313.00	313.00
Meat & bone meal	38.00	38.00	38.00	38.00	38.00	38.00	38.00	38.00	38.00
Limestone	5.00	5.00	5.00	5.00	5.00	5.00	5.00	5.00	5.00
Minerals Zn Se Cr	0.70	0.70	0.70	0.70	0.70	0.70	0.70	0.70	0.70
Sodium bicarbonate	2.36	2.36	2.36	2.36	2.36	2.35	2.35	2.34	2.34
Salt	2.93	2.93	2.93	2.94	2.94	2.94	2.94	2.95	2.95
Vitamin premix [1]	0.96	0.96	0.96	0.96	0.96	0.96	0.96	0.96	0.96
Mineral premix [2]	1.20	1.20	1.20	1.20	1.20	1.20	1.20	1.20	1.20
Enzymes starter	0.40	0.40	0.40	0.40	0.40	0.40	0.40	0.40	0.40
Choline chloride (75%)	0.62	0.62	0.62	0.62	0.62	0.63	0.63	0.63	0.63
L-Lysine sulphate 80	2.79	2.79	2.79	2.77	2.77	2.75	2.75	2.74	2.73
GAA	-	-	-	0.60	0.60	0.60	0.60	0.60	0.60
L-Valine	0.32	0.32	0.32	0.33	0.33	0.33	0.33	0.34	0.34
L-Threonine	1.19	1.19	1.19	1.19	1.19	1.19	1.19	1.20	1.20
MHA-FA	-	3.78	-	-	3.80	-	3.82	-	3.84
DL-Methionine	3.06	-	2.46	3.07	-	3.08	-	3.10	-
Narasin/Nicarbazin	0.63	0.63	0.63	0.63	0.63	0.63	0.63	0.63	0.63
Mycotoxin adsorbent	1.00	1.00	1.00	1.00	1.00	1.00	1.00	1.00	1.00
Formaldehyde	1.00	1.00	1.00	1.00	1.00	1.00	1.00	1.00	1.00
Organic acid	0.30	0.30	0.30	0.30	0.30	0.30	0.30	0.30	0.30
Probiotic	0.40	0.40	0.40	0.40	0.40	0.40	0.40	0.40	0.40
Filler	9.00	8.00	8.00	15.00	15.00	23.00	22.00	30.00	29.00
Total	1000.0	1000.0	1002.0	1000.0	1000.0	1000.0	1000.0	1000.0	1000.0
**Calculated value, %**									
Crude protein	22.35	22.37	22.37	22.47	22.49	22.45	22.47	22.44	22.46
AMEn, kcal/kg	2.999	3.001	3.001	2.976	2.975	2.948	2.950	2.924	2.926
Fat	3.45	3.45	3.45	3.42	3.42	3.39	3.39	3.36	3.36
Crude fibre	2.15	2.15	2.15	2.15	2.14	2.13	2.13	2.12	2.12
Calcium	0.89	0.89	0.89	0.89	0.89	0.89	0.89	0.89	0.89

Available phosphorus	0.47	0.47	0.47	0.47	0.47	0.47	0.47	0.47	0.47
Dig Lys, %	1.20	1.20	1.20	1.20	1.20	1.20	1.20	1.20	1.20
Dig Met, %	0.61	0.60	0.60	0.61	0.61	0.61	0.61	0.61	0.61
Dig Met+Cys, %	0.90	0.90	0.90	0.90	0.90	0.90	0.90	0.90	0.90
Dig Thr, %	0.81	0.81	0.81	0.80	0.80	0.80	0.80	0.80	0.80
Dig Try, %	0.22	0.22	0.22	0.22	0.22	0.22	0.22	0.22	0.22
Dig Arg, %	1.30	1.30	1.30	1.35	1.35	1.35	1.35	1.35	1.35
Dig Iso, %	0.78	0.78	0.78	0.78	0.78	0.78	0.78	0.78	0.78
Dig Leu, %	1.53	1.53	1.53	1.52	1.52	1.52	1.52	1.52	1.52
Dig Val, %	0.91	0.91	0.91	0.91	0.91	0.91	0.91	0.91	0.91
Dig His, %	0.50	0.50	0.50	0.50	0.50	0.50	0.50	0.50	0.50
Dig Fen, %	0.87	0.87	0.87	0.87	0.87	0.87	0.87	0.87	0.87
Dig Gly, %	0.87	0.87	0.87	0.86	0.86	0.86	0.86	0.86	0.86
Met/Lys ratio	0.50	0.50	0.50	0.50	0.50	0.50	0.51	0.51	0.51
M+C/Lys ratio	0.75	0.75	0.75	0.75	0.75	0.75	0.75	0.75	0.75
Thr/Lys ratio	0.67	0.67	0.67	0.67	0.67	0.67	0.67	0.67	0.67
Try/Lys ratio	0.18	0.18	0.18	0.18	0.18	0.18	0.18	0.18	0.18
Arg/Lys ratio	1.08	1.08	1.08	1.12	1.12	1.12	1.12	1.12	1.12
Iso/Lys ratio	0.65	0.65	0.65	0.65	0.65	0.65	0.65	0.65	0.65
Leu/Lys ratio	1.27	1.27	1.27	1.27	1.27	1.27	1.27	1.26	1.26
Val/Lys ratio	0.76	0.76	0.76	0.76	0.76	0.76	0.76	0.76	0.76

Each ton of grower feed contains: [1] 10,000 KIU of vitamin AD3, 4300 KIU of vitamin D3, 70,000 IU of vitamin E, 3600 mg of vitamin K3, 2900 mg of vitamin B1, 6900 mg of vitamin B2, 4000 mg of vitamin B6, 30 mg of vitamin B12, 14,500 mg of pantothenic acid, 60,000 mg of niacin, 1900 mg of folic acid, 270 mg of biotin, and 110 g of BHT; [2] 1000 mg of cobalt (as cobalt sulfate), 15,000 mg of copper (as copper sulfate), 3000 mg of iodine (as calcium iodate monohydrate), 45,000 mg of iron (as ferrous sulfate), 100,000 mg of manganese (as manganese sulfate), 200 mg of selenium (as sodium selenite), and 80,000 mg of zinc (as zinc sulfate).

Table S3. Formulation and nutrient composition of grower diet (days 15–24).

	**Standard**			**−25 kcal/kg**		**−50 kcal/kg**		**−75 kcal/kg**	
**Ingredient, %**	**DLM**	**MHA**	**DL65**	**DLM**	**MHA**	**DLM**	**MHA**	**DLM**	**MHA**
Corn	658.29	658.63	656.98	658.69	659.03	650.69	650.02	641.67	640.99
Soybean meal (46% CP)	281.00	281.00	281.00	281.00	281.00	282.00	282.00	283.00	283.00
Vegetable oil	3.00	3.00	3.00	-	-	-	-	-	-
Meat & bone meal	33.00	33.00	33.00	33.00	33.00	33.00	33.00	34.00	34.00
Limestone	5.00	5.00	5.00	5.00	5.00	5.00	5.00	5.00	5.00

Minerals Zn Se Cr	0.70	0.70	0.70	0.70	0.70	0.70	0.70	0.70	0.70
Sodium bicarbonate	1.89	1.89	1.89	1.89	1.89	1.89	1.89	1.88	1.88
Salt	2.95	2.95	2.95	2.95	2.95	2.95	2.95	2.96	2.96
Vitamin premix [1]	0.80	0.80	0.80	0.80	0.80	0.80	0.80	0.80	0.80
Mineral premix [2]	1.20	1.20	1.20	1.20	1.20	1.20	1.20	1.20	1.20
Enzyme grower	0.40	0.40	0.40	0.40	0.40	0.40	0.40	0.40	0.40
Choline chloride (75%)	0.57	0.57	0.57	0.57	0.57	0.57	0.57	0.58	0.58
L-Lysine sulphate 80	2.89	2.89	2.89	2.89	2.89	2.87	2.87	2.86	2.86
GAA	-	-	-	0.60	0.60	0.60	0.60	0.60	0.60
L-Valine	0.23	0.23	0.23	0.23	0.23	0.23	0.23	0.24	0.24
L-Threonine	1.12	1.12	1.12	1.12	1.12	1.13	1.13	1.13	1.13
MHA-FA	-	3.50	3.50	-	3.50	-	3.52	-	3.54
DL-Methionine	2.84	-	2.28	2.84	-	2.85	-	2.86	-
Narasin/Nicarbazin	0.63	0.63	-	0.63	0.63	0.63	0.63	0.63	0.63
Mycotoxin adsorbent	1.00	1.00	1.00	1.00	1.00	1.00	1.00	1.00	1.00
Organic acid	0.30	0.30	0.30	0.30	0.30	0.30	0.30	0.30	0.30
Probiotic	0.20	0.20	0.20	0.20	0.20	0.20	0.20	0.20	0.20
Filler	2.00	1.00	1.00	4.00	3.00	11.00	11.00	18.00	18.00
Total	1000.0	1000.0	1000.0	1000.0	1000.0	1000.0	1000.0	1000.0	1000.0
**Calculated value, %**									
Crude protein	20.93	20.95	20.95	21.07	21.09	21.06	21.07	21.09	21.10
AMEn, kcal/kg	3.090	3.093	3.093	3.065	3.068	3.041	3.040	3.015	3.014
Fat	3.79	3.79	3.79	3.49	3.49	3.46	3.46	3.44	3.44
Crude fibre	2.11	2.11	2.11	2.11	2.11	2.10	2.10	2.09	2.09
Calcium	0.83	0.83	0.83	0.83	0.83	0.83	0.83	0.84	0.84
Available phosphorus	0.44	0.44	0.44	0.44	0.44	0.44	0.44	0.44	0.44
Dig Lys, %	1.12	1.12	1.12	1.12	1.12	1.12	1.12	1.12	1.12
Dig Met, %	0.57	0.57	0.57	0.57	0.57	0.57	0.57	0.57	0.57
Dig Met+Cys, %	0.85	0.85	0.85	0.85	0.85	0.85	0.85	0.85	0.85
Dig Thr, %	0.75	0.75	0.75	0.75	0.75	0.75	0.75	0.75	0.75
Dig Try, %	0.20	0.20	0.20	0.20	0.20	0.20	0.20	0.20	0.20
Dig Arg, %	1.21	1.21	1.21	1.25	1.25	1.25	1.25	1.26	1.26
Dig Iso, %	0.73	0.73	0.73	0.73	0.73	0.73	0.73	0.73	0.73
Dig Leu, %	1.46	1.46	1.46	1.46	1.46	1.45	1.45	1.45	1.45
Dig Val, %	0.85	0.85	0.85	0.85	0.85	0.85	0.85	0.85	0.85

Dig His, %	0.47	0.47	0.47	0.47	0.47	0.47	0.47	0.47	0.47
Dig Fen, %	0.82	0.82	0.82	0.82	0.82	0.82	0.82	0.82	0.82
Dig Gly, %	0.80	0.80	0.80	0.80	0.80	0.80	0.80	0.81	0.81
Met/Lys ratio	0.51	0.51	0.51	0.51	0.51	0.51	0.51	0.51	0.51
M+C/Lys ratio	0.76	0.76	0.76	0.76	0.76	0.76	0.76	0.76	0.76
Thr/Lys ratio	0.67	0.67	0.67	0.67	0.67	0.67	0.67	0.67	0.67
Try/Lys ratio	0.18	0.18	0.18	0.18	0.18	0.18	0.18	0.18	0.18
Arg/Lys ratio	1.08	1.08	1.08	1.12	1.12	1.12	1.12	1.12	1.12
Iso/Lys ratio	0.65	0.65	0.65	0.65	0.65	0.65	0.65	0.65	0.65
Leu/Lys ratio	1.30	1.30	1.30	1.30	1.30	1.30	1.30	1.30	1.30
Val/Lys ratio	0.76	0.76	0.76	0.76	0.76	0.76	0.76	0.76	0.76

Each ton of grower feed contains: [1] 10,000 KIU of vitamin AD3, 4300 KIU of vitamin D3, 70,000 IU of vitamin E, 3600 mg of vitamin K3, 2900 mg of vitamin B1, 6900 mg of vitamin B2, 4000 mg of vitamin B6, 30 mg of vitamin B12, 14,500 mg of pantothenic acid, 60,000 mg of niacin, 1900 mg of folic acid, 270 mg of biotin, and 110 g of BHT; [2] 1000 mg of cobalt (as cobalt sulfate), 15,000 mg of copper (as copper sulfate), 3000 mg of iodine (as calcium iodate monohydrate), 45,000 mg of iron (as ferrous sulfate), 100,000 mg of manganese (as manganese sulfate), 200 mg of selenium (as sodium selenite), and 80,000 mg of zinc (as zinc sulfate).

Table S4. Formulation and nutrient composition of finisher I diet (days 25–35).

	**Standard**			**−25 kcal/kg**		**−50 kcal/kg**		**−75 kcal/kg**	
Ingredient, %	**DLM**	**MHA**	**DL65**	**DLM**	**MHA**	**DLM**	**MHA**	**DLM**	**MHA**
Corn	702.11	701.57	702.57	694.51	693.96	686.50	685.94	678.49	677.93
Soybean meal (46% CP)	247.00	247.00	247.00	248.00	248.00	249.00	249.00	250.00	250.00
Meat & bone meal	30.00	30.00	30.00	30.00	30.00	30.00	30.00	30.00	30.00
Limestone	5.00	5.00	5.00	5.00	5.00	5.00	5.00	5.00	5.00
Salt	2.93	2.93	2.93	2.93	2.93	2.94	2.94	2.95	2.95
Minerals Zn Se Cr	0.70	0.70	0.70	0.70	0.70	0.70	0.70	0.70	0.70
Sodium bicarbonate	1.64	1.64	1.64	1.63	1.63	1.62	1.62	1.61	1.61
Vitamin premix [1]	0.80	0.80	0.80	0.80	0.80	0.80	0.80	0.80	0.80
Mineral premix [2]	1.20	1.20	1.20	1.20	1.20	1.20	1.20	1.20	1.20
Enzyme grower	0.40	0.40	0.40	0.40	0.40	0.40	0.40	0.40	0.40
Choline chloride (75%)	0.61	0.61	0.61	0.61	0.61	0.62	0.62	0.62	0.62
L-Lysine sulphate 80	2.60	2.60	2.60	2.59	2.59	2.57	2.57	2.55	2.55
GAA	-	-	-	0.60	0.60	0.60	0.60	0.60	0.60
L-Valine	0.11	0.11	0.11	0.12	0.12	0.12	0.12	0.13	0.13
L-Threonine	0.83	0.83	0.83	0.83	0.83	0.84	0.84	0.84	0.84

MHA-FA	-	2.86	-	-	2.88	-	2.90	-	2.92
DL-Methionine	2.32	-	1.86	2.33	-	2.34	-	2.36	-
Monensin	0.25	0.25	0.25	0.25	0.25	0.25	0.25	0.25	0.25
Mycotoxin adsorbent	1.00	1.00	1.00	1.00	1.00	1.00	1.00	1.00	1.00
Organic acid	0.30	0.30	0.30	0.30	0.30	0.30	0.30	0.30	0.30
Probiotic	0.20	0.20	0.20	0.20	0.20	0.20	0.20	0.20	0.20
Filler	-	-	-	6.00	6.00	13.00	13.00	20.00	20.00
Total	1000.0	1000.0	1000.0	1000.0	1000.0	1000.0	1000.0	1000.0	1000.0
**Calculated value, %**									
Crude protein	19.42	19.43	19.43	19.55	19.56	19.54	19.55	19.53	19.54
AMEn, kcal/kg	3.120	3.119	3.119	3.097	3.096	3.072	3.072	3.048	3.047
Fat	3.46	3.44	3.44	3.53	3.53	3.50	3.50	3.47	3.47
Crude fibre	2.10	2.09	2.09	2.05	2.05	2.04	2.04	2.03	2.03
Calcium	0.83	0.84	0.84	0.78	0.78	0.78	0.78	0.78	0.78
Available phosphorus	0.44	0.44	0.44	0.42	0.42	0.42	0.42	0.42	0.42
Dig Lys, %	1.02	1.02	1.02	1.02	1.02	1.02	1.02	1.02	1.02
Dig Met, %	0.51	0.50	0.50	0.51	0.51	0.51	0.51	0.51	0.51
Dig Met+Cys, %	0.78	0.77	0.77	0.78	0.78	0.78	0.78	0.78	0.78
Dig Thr, %	0.68	0.68	0.68	0.68	0.68	0.68	0.68	0.68	0.68
Dig Try, %	0.19	0.19	0.19	0.19	0.19	0.19	0.19	0.19	0.19
Dig Arg, %	1.25	1.26	1.26	1.16	1.15	1.16	1.16	1.16	1.16
Dig Iso, %	0.73	0.73	0.73	0.67	0.67	0.67	0.67	0.67	0.67
Dig Leu, %	1.45	1.45	1.45	1.38	1.38	1.37	1.37	1.37	1.37
Dig Val, %	0.85	0.85	0.85	0.79	0.79	0.79	0.78	0.79	0.79
Dig His, %	0.47	0.47	0.47	0.44	0.44	0.44	0.44	0.44	0.44
Dig Fen, %	0.82	0.82	0.82	0.76	0.76	0.76	0.76	0.76	0.76
Dig Gly, %	0.80	0.81	0.81	0.74	0.74	0.74	0.74	0.74	0.74
Met/Lys ratio	0.51	0.51	0.51	0.50	0.50	0.50	0.50	0.50	0.50
M+C/Lys ratio	0.76	0.76	0.76	0.76	0.76	0.76	0.76	0.76	0.76
Thr/Lys ratio	0.67	0.67	0.67	0.67	0.67	0.67	0.67	0.67	0.67
Try/Lys ratio	0.18	0.18	0.18	0.18	0.18	0.18	0.18	0.18	0.18
Arg/Lys ratio	1.12	1.12	1.12	1.13	1.13	1.13	1.13	1.13	1.13
Iso/Lys ratio	0.65	0.65	0.65	0.66	0.66	0.66	0.66	0.66	0.66
Leu/Lys ratio	1.30	1.30	1.30	1.35	1.35	1.35	1.35	1.35	1.35
Val/Lys ratio	0.76	0.76	0.76	0.77	0.77	0.77	0.77	0.77	0.77

Each ton of grower II feed contains: [1] 10,000 KIU of vitamin AD3, 4300 KIU of vitamin D3, 70,000 IU of vitamin E, 3600 mg of vitamin K3, 2900 mg of vitamin B1, 6900 mg of vitamin B2, 4000 mg of vitamin B6, 30 mg of vitamin B12, 14,500 mg of pantothenic acid, 60,000 mg of niacin, 1900 mg of folic acid, 270 mg of biotin, and 110 g of BHT; [2] 1000 mg of cobalt (as cobalt sulfate), 15,000 mg of copper (as copper sulfate), 3000 mg of iodine (as calcium iodate monohydrate), 45,000 mg of iron (as ferrous sulfate), 100,000 mg of manganese (as manganese sulfate), 200 mg of selenium (as sodium selenite), and 80,000 mg of zinc (as zinc sulfate).

Table S5. Formulation and nutrient composition of finisher II diet (days 36–40).

	Standard			−25 kcal/kg		−50 kcal/kg		−75 kcal/kg	
	**DLM**	**MHA**	**DL65**	**DLM**	**MHA**	**DLM**	**MHA**	**DLM**	**MHA**
Ingredient, %									
Corn	737.29	736.84	737.67	728.70	728.24	720.68	720.22	712.67	712.20
Soybean meal (46% CP)	210.00	210.00	210.00	211.00	211.00	212.00	212.00	213.00	213.00
Meat & bone meal	27.00	27.00	27.00	27.00	27.00	27.00	27.00	27.00	27.00
Limestone	5.00	5.00	5.00	5.00	5.00	5.00	5.00	5.00	5.00
Salt	2.91	2.91	2.91	2.91	2.91	2.92	2.92	2.93	2.93
Minerals Zn Se Cr	0.70	0.70	0.70	0.70	0.70	0.70	0.70	0.70	0.70
Sodium bicarbonate	1.38	1.38	1.38	1.38	1.38	1.37	1.37	1.36	1.36
Mineral premix [2]	1.20	1.20	1.20	1.20	1.20	1.20	1.20	1.20	1.20
Vitamin premix [1]	0.64	0.64	0.64	0.64	0.64	0.64	0.64	0.64	0.64
Enzyme grower	0.40	0.40	0.40	0.40	0.40	0.40	0.40	0.40	0.40
Choline chloride (75%)	0.68	0.68	0.68	0.68	0.68	0.68	0.68	0.68	0.68
L-Lysine sulphate 80	2.59	2.59	2.59	2.57	2.57	2.56	2.56	2.54	2.54
GAA	-	-	-	0.60	0.60	0.60	0.60	0.60	0.60
L-Valine	0.10	0.10	0.10	0.10	0.10	0.11	0.11	0.12	0.12
L-Threonine	0.70	0.70	0.70	0.70	0.70	0.70	0.70	0.71	0.71
MHA-FA	-	2.36	-	-	2.38	-	2.40	-	2.42
DL-Methionine	1.91	-	1.53	1.92	-	1.94	-	1.95	-
Organic acid	0.30	0.30	0.30	0.30	0.30	0.30	0.30	0.30	0.30
Probiotic	0.20	0.20	0.20	0.20	0.20	0.20	0.20	0.20	0.20
Filler	7.00	7.00	7.00	14.00	14.00	21.00	21.00	28.00	28.00
Total	1000.0	1000.0	1000.0	1000.0	1000.0	1000.0	1000.0	1000.0	1000.0
**Calculated value, %**									
Crude protein	17.70	17.70	17.70	17.81	17.82	17.80	17.81	17.79	17.80
AMEn, kcal/kg	3.153	3.152	3.152	3.126	3.125	3.102	3.101	3.077	3.077
Fat	3.58	3.58	3.58	3.55	3.55	3.52	3.52	3.49	3.49

Crude fibre	1.98	1.98	1.98	1.97	1.97	1.96	1.96	1.95	1.95
Calcium	0.74	0.74	0.74	0.74	0.74	0.74	0.74	0.74	0.74
Available phosphorus	0.40	0.40	0.40	0.40	0.40	0.40	0.40	0.40	0.40
Dig Lys, %	0.92	0.92	0.92	0.92	0.92	0.92	0.92	0.92	0.92
Dig Met, %	0.45	0.45	0.45	0.45	0.45	0.45	0.45	0.45	0.45
Dig Met+Cys, %	0.70	0.70	0.70	0.70	0.70	0.70	0.70	0.70	0.70
Dig Thr, %	0.62	0.62	0.62	0.62	0.62	0.62	0.62	0.62	0.62
Dig Try, %	0.17	0.17	0.17	0.17	0.17	0.17	0.17	0.17	0.17
Dig Arg, %	0.99	0.99	0.99	1.04	1.04	1.04	1.04	1.04	1.04
Dig Iso, %	0.61	0.61	0.61	0.61	0.61	0.61	0.61	0.61	0.61
Dig Leu, %	1.29	1.29	1.29	1.28	1.28	1.28	1.28	1.28	1.28
Dig Val, %	0.72	0.72	0.72	0.72	0.72	0.72	0.72	0.72	0.72
Dig His, %	0.41	0.41	0.41	0.41	0.41	0.41	0.41	0.41	0.41
Dig Fen, %	0.69	0.69	0.69	0.69	0.69	0.69	0.69	0.69	0.69
Dig Gly, %	0.67	0.67	0.67	0.67	0.67	0.67	0.67	0.67	0.67
Met/Lys ratio	0.49	0.48	0.48	0.49	0.49	0.49	0.49	0.49	0.49
M+C/Lys ratio	0.76	0.76	0.76	0.76	0.76	0.76	0.76	0.76	0.76
Thr/Lys ratio	0.67	0.67	0.67	0.67	0.67	0.67	0.67	0.67	0.67
Try/Lys ratio	0.18	0.18	0.18	0.18	0.18	0.18	0.18	0.18	0.18
Arg/Lys ratio	1.08	1.08	1.08	1.13	1.13	1.13	1.13	1.13	1.13
Iso/Lys ratio	0.66	0.66	0.66	0.66	0.66	0.66	0.66	0.66	0.66
Leu/Lys ratio	1.40	1.40	1.40	1.39	1.39	1.39	1.39	1.39	1.39
Val/Lys ratio	0.78	0.78	0.78	0.78	0.78	0.78	0.78	0.78	0.78

Each kilogram of finisher feed contains: [1] 8000 KIU of vitamin AD3, 3440 KIU of vitamin D3, 56,000 IU of vitamin E, 2880 mg of vitamin K3, 2320 mg of vitamin B1, 5520 mg of vitamin B2, 3200 mg of vitamin B6, 24 mg of vitamin B12, 11,600 mg of pantothenic acid, 48,000 mg of niacin, 1520 mg of folic acid, 216 mg of biotin, and 88 g of BHT; [2] 800 mg of cobalt (as cobalt sulfate), 12,000 mg of copper (as copper sulfate), 2400 mg of iodine (as calcium iodate monohydrate), 36,000 mg of iron (as ferrous sulfate), 80,000 mg of manganese (as manganese sulfate), 160 mg of selenium (as sodium selenite), and 64,000 mg of zinc (as zinc sulfate).

Table S6. Analyzed nutrient compositions of pre-starter diet (days 0–7).

Analyzed Value as-Fed Basis %	Standard			−25 kcal/kg		−50 kcal/kg		−75 kcal/kg	
	DL-MET	MHA-FA	DL-Met65	DL-MET	MHA-FA	DL-MET	MHA-FA	DL-MET	MHA-FA
Lysine total	1.36	1.38	1.40	1.37	1.35	1.31	1.34	1.31	1.35
Methionine total	0.61	0.35	0.56	0.57	0.37	0.60	0.36	0.59	0.40
Methionine + Cysteine total	0.95	0.68	0.90	0.91	0.71	0.94	0.72	0.95	0.75
MET SUPPLEMENTED	0.31	0.05	0.23	0.28	0.06	0.29	0.05	0.28	0.08
MHA-FA SUPPLEMENTED	0.03	0.37	0.05	0.03	0.39	0.04	0.38	0.04	0.37
LYS SUPPLEMENTED	0.21	0.21	0.21	0.21	0.22	0.20	0.20	0.20	0.22
THR SUPPLEMENTED	0.13	0.13	0.13	0.12	0.14	0.13	0.13	0.13	0.13
VAL SUPPLEMENTED	0.05	<0.020	0.05	0.05	<0.020	<0.020	<0.020	<0.020	0.06
GAA SUPPLEMENTED	not detectable	not detectable	not detectable	379	493	523	479	500	497

Table S7. Analyzed nutrient compositions of starter diet (days 8–14).

Analyzed Value as-Fed Basis %	Standard			−25 kcal/kg		−50 kcal/kg		−75 kcal/kg	
	DL-MET	MHA-FA	DL-Met65	DL-MET	MHA-FA	DL-MET	MHA-FA	DL-MET	MHA-FA
Lysine total	1.25	1.24	1.23	1.28	1.30	1.24	1.33	1.27	1.26
Methionine total	0.57	0.35	0.49	0.55	0.34	0.56	0.35	0.55	0.34
Methionine + Cysteine total	0.93	0.69	0.84	0.87	0.66	0.87	0.68	0.87	0.66
MET SUPPLEMENTED	0.26	0.04	0.19	0.25	0.05	0.26	0.04	0.26	0.05
MHA-FA SUPPLEMENTED	0.03	0.34	0.04	0.03	0.33	0.03	0.34	0.03	0.32
LYS SUPPLEMENTED	0.18	0.19	0.19	0.18	0.22	0.21	0.20	0.19	0.18
THR SUPPLEMENTED	0.10	0.10	0.09	0.10	0.10	0.10	0.10	0.10	0.10
VAL SUPPLEMENTED	0.04	0.04	<0.020	0.03	0.04	0.04	<0.020	0.04	<0.020
GAA SUPPLEMENTED	not detectable	not detectable	not detectable	510	512	546	516	553	502

Table S8. Analyzed nutrient compositions of grower diet (days 15–24).

Analyzed Value as-Fed Basis %	Standard			−25 kcal/kg		−50 kcal/kg		−75 kcal/kg	
	DL-MET	MHA-FA	DL-Met65	DL-MET	MHA-FA	DL-MET	MHA-FA	DL-MET	MHA-FA
Lysine total	1.26	1.24	1.20	1.23	1.22	1.24	1.26	1.23	1.22
Methionine total	0.53	0.33	0.49	0.54	0.32	0.51	0.33	0.53	0.34
Methionine + Cysteine total	0.85	0.64	0.80	0.86	0.64	0.83	0.65	0.85	0.65
MET SUPPLEMENTED	0.25	0.03	0.20	0.24	0.03	0.23	0.03	0.25	0.04
MHA-FA SUPPLEMENTED	0.02	0.31	0.02	0.02	0.30	0.03	0.31	0.02	0.30
LYS SUPPLEMENTED	0.20	0.20	0.19	0.19	0.21	0.21	0.21	0.20	0.19
THR SUPPLEMENTED	0.10	0.09	0.09	0.09	0.09	0.08	0.10	0.09	0.09
VAL SUPPLEMENTED	0.03	0.03	0.03	0.03	0.03	0.03	0.03	0.03	0.03
GAA SUPPLEMENTED	not detectable	not detectable	not detectable	563	566	643	621	616	563

Table S9. Analyzed nutrient compositions of finisher I diet (days 25–35).

Analyzed Value as-Fed Basis %	Standard			−25 kcal/kg		−50 kcal/kg		−75 kcal/kg	
	DL-MET	MHA-FA	DL-Met65	DL-MET	MHA-FA	DL-MET	MHA-FA	DL-MET	MHA-FA
Lysine total	1.18	1.08	1.11	1.17	1.01	1.13	1.06	1.10	1.08
Methionine total	0.40	0.28	0.37	0.42	0.29	0.40	0.30	0.39	0.31
Methionine + Cysteine total	0.69	0.57	0.66	0.70	0.57	0.70	0.59	0.67	0.60
MET SUPPLEMENTED	0.14	0.02	0.11	0.14	0.02	0.12	0.02	0.12	0.04
MHA-FA SUPPLEMENTED	0.02	0.24	0.02	0.01	0.25	0.01	0.25	0.02	0.23
LYS SUPPLEMENTED	0.24	0.17	0.21	0.23	0.13	0.21	0.13	0.21	0.14
THR SUPPLEMENTED	0.07	0.07	0.07	0.07	0.07	0.07	0.07	0.06	0.07
VAL SUPPLEMENTED	<0.02	<0.02	<0.02	<0.02	<0.02	<0.02	<0.02	<0.02	<0.02
GAA SUPPLEMENTED	not detectable	not detectable	not detectable	587	635	615	600	580	625

Table S10. Analyzed nutrient compositions of finisher II diet (days 36–40).

Analyzed Value as-Fed Basis %	Standard			−25 kcal/kg		−50 kcal/kg		−75 kcal/kg	
	DL-MET	MHA-FA	DL-Met65	DL-MET	MHA-FA	DL-MET	MHA-FA	DL-MET	MHA-FA
Lysine total	0.97	1.00	1.00	1.02	1.02	1.00	1.01	1.01	1.02
Methionine total	0.40	0.27	0.39	0.43	0.28	0.41	0.28	0.41	0.28
Methionine + Cysteine total	0.67	0.55	0.67	0.71	0.56	0.68	0.56	0.69	0.56
MET SUPPLEMENTED	0.15	0.02	0.13	0.16	0.02	0.17	0.02	0.17	0.02
MHA-FA SUPPLEMENTED	0.04	0.20	0.02	0.01	0.20	0.02	0.20	0.02	0.21
LYS SUPPLEMENTED	0.18	0.17	0.18	0.18	0.20	0.20	0.19	0.18	0.18
THR SUPPLEMENTED	0.06	0.06	0	0.06	0.07	0.06	0.06	0.07	0.07
VAL SUPPLEMENTED	<0.02	<0.02	<0.02	<0.02	<0.02	<0.02	<0.02	<0.02	<0.02
GAA SUPPLEMENTED	not detectable	not detectable	not detectable	501	650	616	635	650	617
